# A comprehensive tRNA pseudouridine map uncovers targets dependent on human stand-alone pseudouridine synthases

**DOI:** 10.1038/s41556-025-01803-w

**Published:** 2025-10-24

**Authors:** Haiqi Xu, Linzhen Kong, Mengjie Li, Giuseppina Pisignano, Jingfei Cheng, Feng Feng, Parinaz Mehdipour, Chun-Xiao Song

**Affiliations:** 1 Ludwig Institute for Cancer Research, Nuffield Department of Medicine, University of Oxford, Oxford, UK; 2 Target Discovery Institute, Nuffield Department of Medicine, University of Oxford, Oxford, UK

**Keywords:** RNA, RNA

## Abstract

Pseudouridine (Ψ) is one of the most abundant RNA modifications in human cells, introduced post-transcriptionally by pseudouridine synthases (PUS). Despite its prevalence, the biological functions of Ψ remain poorly understood, largely due to the limited knowledge linking specific PUS enzymes to their targets. Here, to address this gap, we systematically knocked out or knocked down nine stand-alone PUS in HCT116 cells and mapped their Ψ profiles using 2-bromoacrylamide-assisted cyclization sequencing. Through this approach, we uncovered previously unknown targets of several PUS enzymes, including RPUSD1, RPUSD2, PUS3, PUSL1 and PUS7L. In addition, we revealed that TRUB1 and PUS10 function redundantly to catalyse the highly conserved Ψ55 modification in cytosolic tRNAs. Intriguingly, we found that RPUSD3 and TRUB2 do not exhibit noticeable enzymatic activities in human cells. By integrating these findings with earlier results for TRUB1, PUS7 and PUS1, we constructed a comprehensive map of stand-alone PUS-dependent Ψ modifications across human tRNAs. Using this map, we further demonstrated that different PUS enzymes introduce Ψ modifications at distinct stages of pre-tRNA processing.

## Abstract (web summary)

Xu, Kong, Li, Pisignano and colleagues provide a map of pseudouridine sites in human noncoding RNA. Using a systematic knockout approach, they associate each pseudouridine site with the activity of a specific pseudouridine synthase.

## Main

Pseudouridine (Ψ) is the first identified and the most abundant RNA modification, predominantly found in noncoding RNAs (ncRNAs), including ribosomal RNA (rRNAs), small nuclear RNAs (snRNAs) and transfer RNAs (tRNAs)^1,2^. Ψ plays important roles in translation, splicing and RNA stability^3^. Ψ is deposited by a class of enzymes called pseudouridine synthases (PUS)^4^. A total of 13 PUS enzymes (PUS1, PUSL1, PUS3, PUS7, PUS7L, PUS10, TRUB1, TRUB2, RPUSD1–4 and DKC1) have been annotated in the human genome, which are associated with various genetic diseases and cancer^3^. Therefore, there is a clear need to elucidate their enzymatic targets within the cells.

Based on the catalytic mechanisms, there are two types of human PUS. DKC1 utilizes box H/ACA small nucleolar RNAs (snoRNAs) to catalyse pseudouridylation, which predominantly works on cytosolic rRNAs and spliceosomal snRNAs^5^. By comparison, the other 12 human PUS are considered ‘stand-alone’ enzymes. Most existing studies on human stand-alone PUS have focused on those conserved from their corresponding *Escherichia coli* and *Saccharomyces* *cerevisiae* homologues, including TRUB1, PUS7 and PUS1 (Supplementary Table 1). TRUB1 is found to modify the highly conserved Ψ55 sites in tRNA T-loops^6,7^ and serves as the major PUS for mRNA pseudouridylation with a GUΨCNA (N = A, C, G or U) motif for tRNA mimicry^8,9^. PUS7 is known to catalyse the Ψ13 site in the tRNA D-arm^10,11^, as well as the Ψ35 site in the anticodon^12^, with a UNΨAR (N = A, C, G or U; R = A or G) sequence motif^13^. Unlike TRUB1 and PUS7, PUS1 modifies consecutive Ψ27–28 sites in human tRNAs^14,15^, which lacks a clear sequence motif but instead relies on structural motifs^16^.

Traditionally, the detection of Ψ has relied heavily on *N*-cyclohexyl-*N*′-(2-morpholinoethyl) carbodiimide methyl-*p*-toluenesulfonate (CMC) chemistry^17^. However, this method is known to suffer from high background noise, difficulty in mapping Ψ at single-base resolution, limited ability to quantify Ψ stoichiometry and failure to detect densely modified Ψ sites. To overcome these limitations, we recently developed 2-bromoacrylamide-assisted cyclization sequencing (BACS) for direct, quantitative and base-resolution sequencing of Ψ through inducing Ψ-to-C mutations^18^. BACS outperformed traditional CMC^8,13^ and bisulfite-based methods^19,20^ in terms of higher sensitivity, considerably lower background noise, more precise determination of Ψ positions, more accurate quantification of Ψ stoichiometry and more robust detection of densely modified Ψ sites. We have applied it to study the targets of human TRUB1, PUS7 and PUS1 in HeLa cells demonstrated that these three PUS enzymes have broader target ranges than previously thought^18^ (Supplementary Table 1). However, the targets of the other nine human stand-alone PUS have not been thoroughly studied^4,21^.

**Extended Data Fig. 1:**
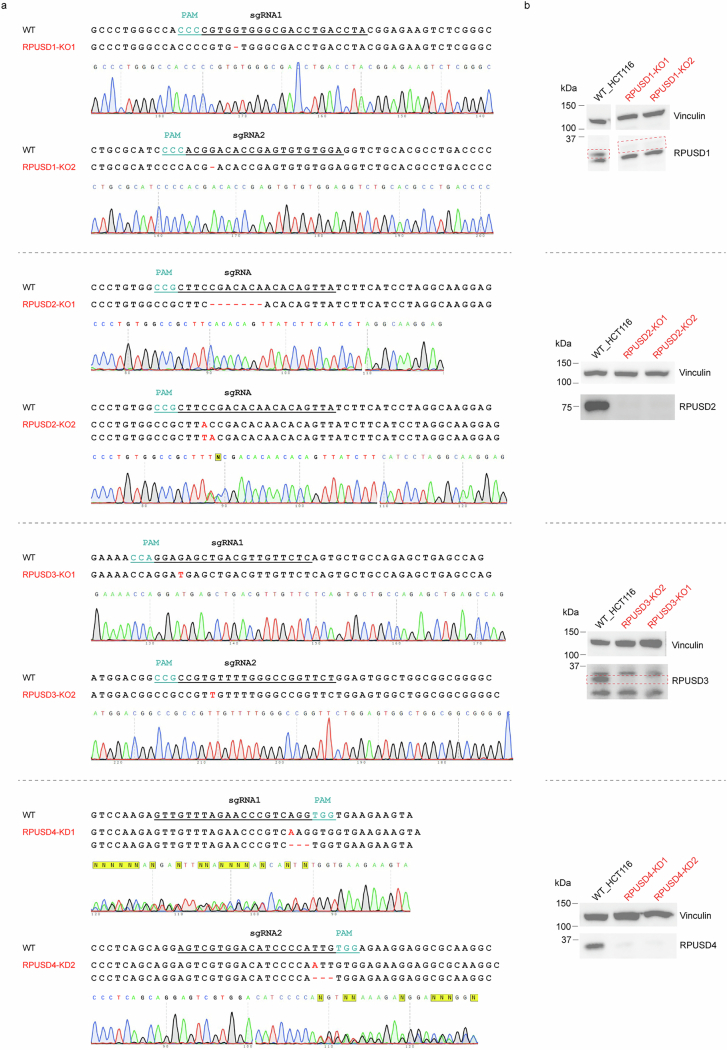
Validation of RPUSD1–3 KO and RPUSD4-KD cells. **(a)** Sanger sequencing of RPUSD1–3 KO and RPUSD4-KD cells, illustrating CRISPR-induced indels in HCT116 cells. **(b)** Representative immunoblots from two independent experiments depict the efficiency of two distinct monoclonal KOs or KDs in HCT116 cells. Anti-Vinculin served as a loading control. Source data

**Extended Data Fig. 2:**
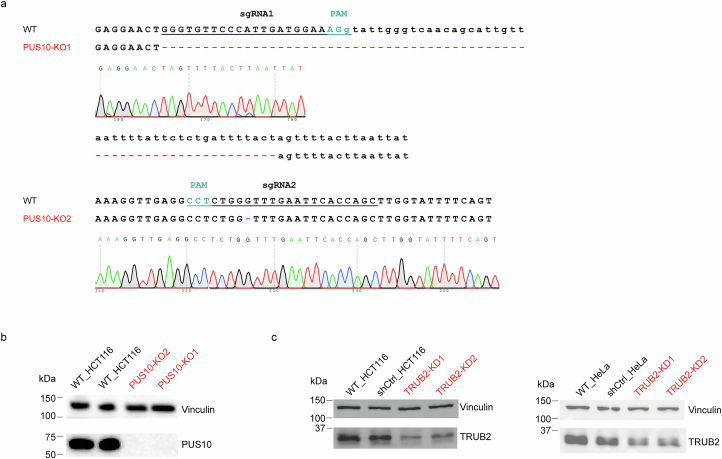
Validation of PUS10-KO and TRUB2-KD cells. **(a)** Sanger sequencing of PUS10-KO cells, illustrating CRISPR-induced indels in HCT116 cells. **(b)** Representative immunoblots from two independent experiments depict the efficiency of two distinct monoclonal PUS10-KOs in HCT116 cells. Anti-Vinculin served as a loading control. **(c)** Representative immunoblots from two independent experiments depict the efficiency of two distinct shRNA TRUB2-KDs in HCT116 and HeLa cells. Anti-Vinculin served as a loading control. Source data

**Extended Data Fig. 3:**
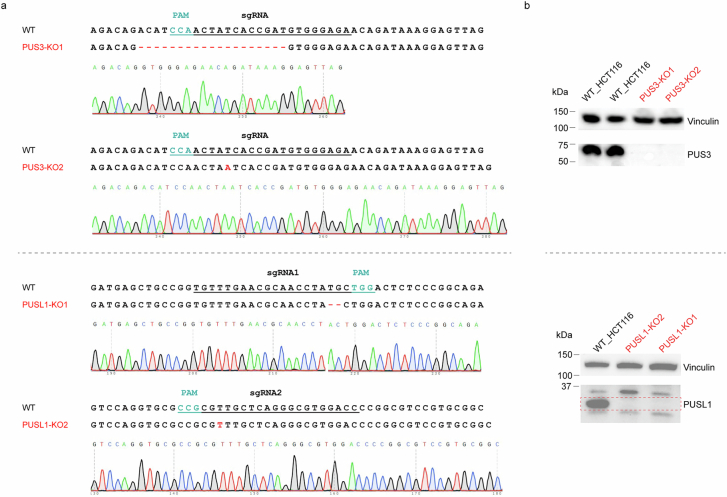
Validation of PUS3-KO and PUSL1-KO cells. **(a)** Sanger sequencing of PUS3-KO and PUSL1-KO cells, illustrating CRISPR-induced indels in HCT116 cells. **(b)** Representative immunoblots from two independent experiments depict the efficiency of two distinct monoclonal KOs in HCT116 cells. Anti-Vinculin served as a loading control. Source data

**Extended Data Fig. 4:**
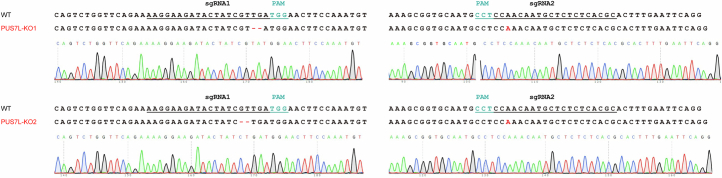
Validation of PUS7L-KO cell lines. Sanger sequencing of PUS7L-KO cells illustrating CRISPR-induced indels in HCT116 cells.

To build a comprehensive stand-alone PUS-dependent Ψ map, we individually knocked out seven PUS enzymes (PUS3, PUS7L, PUS10, PUSL1 and RPUSD1–3) and knocked down two PUS enzymes (RPUSD4 and TRUB2) in HCT116 colorectal cancer cells. We then used BACS to detect Ψ across ncRNAs (Extended Data Figs. 1–4). By comparing the results with those from wild-type (WT) cells, we demonstrated that RPUSD1 and RPUSD2 are responsible for several previously unknown Ψ targets in tRNAs, while RPUSD4 predominantly works on 16S mitochondrial rRNA (mt-rRNA). In addition, we found that TRUB1 and PUS10 are redundant for Ψ55 in human cytosolic tRNAs (cy-tRNAs), while PUS3 and PUSL1 catalyse Ψ38–40 sites in cy-tRNAs and mitochondrial tRNAs (mt-tRNAs), respectively. In stark contrast to PUS7, PUS7L displays unique activities on cy-tRNA variable loops. Interestingly, two PUS enzymes (RPUSD3 and TRUB2) do not present noticeable enzymatic activities on their predicted rRNA or tRNA targets. Compared with their *E. coli* and *S. cerevisiae* homologues, human PUS enzymes generally exhibit more diverse enzymatic activities. Finally, benefitting from this comprehensive stand-alone PUS-dependent tRNA Ψ map, we demonstrated that human PUS enzymes install Ψ at various stages of precursor tRNA (pre-tRNA) processing.

## Results

### RPUSD1–4 exhibit distinct enzymatic activities

**Extended Data Fig. 5:**
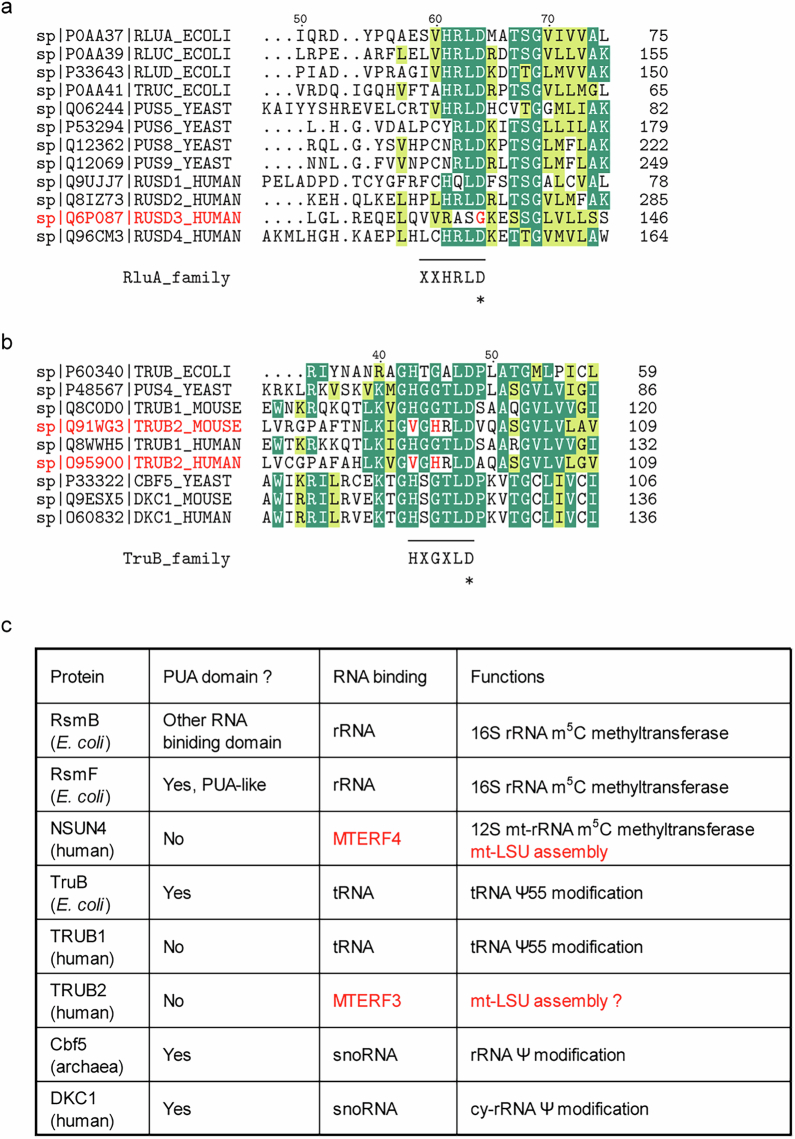
RPUSD3 and TRUB2 might not be active PUS enzymes in vivo. **(a)** Sequence alignments of RPUSD3 with other members of the RluA family. Deviations from the consensus motif are highlighted in red, and the catalytic residue is marked with a star. **(b)** Sequence alignments of TRUB2 with other members of the TruB family. Deviations from the consensus motif are highlighted in red, and the catalytic residue is marked with a star. **(c)** Comparison of TRUB2 with relevant RNA-modifying enzymes.

Human RPUSD1–4 enzymes belong to the RluA family, whose ancestral bacterial RluA presents dual activities on both rRNAs and tRNAs^22^ (Extended Data Fig. 5a). However, the targets of human RPUSD1–4 have not been extensively studied.

**Fig. 1:**
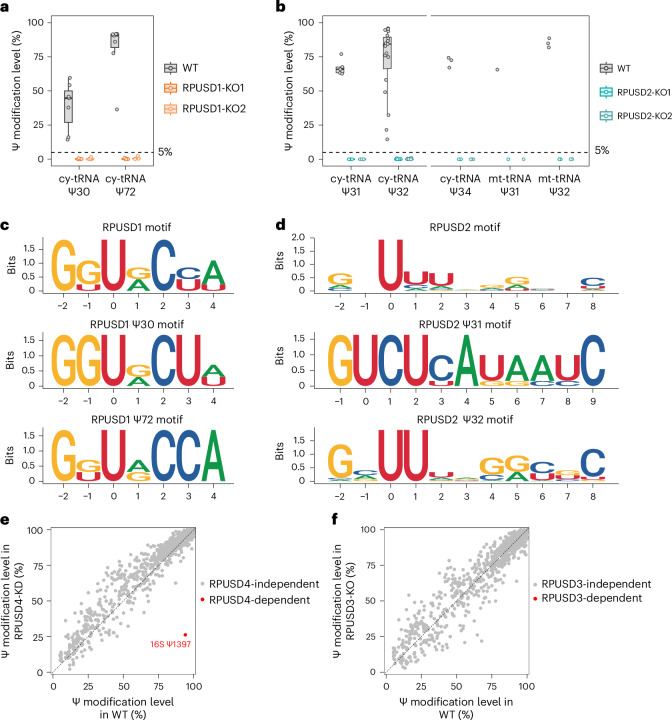
Characterization of human RPUSD1–4 enzymatic activities. **a**, Comparison of the modification levels of Ψ sites at selected positions of human tRNAs upon RPUSD1 depletion. Box plots visualize all Ψ sites at each position; boxes represent the 25th to 75th percentiles with a line at the median; whiskers correspond to 1.5 times the interquartile range (cy-tRNA: Ψ30, *n* = 7 Ψ sites; Ψ72, *n* = 7 Ψ sites). **b**, Comparison of the modification levels of Ψ sites at selected positions of human tRNAs upon RPUSD2 depletion. Box plots visualize all Ψ sites at each position; boxes represent the 25th to 75th percentiles with a line at the median; whiskers correspond to 1.5 times the interquartile range (cy-tRNA: Ψ31, *n* = 5; Ψ32, *n* = 19; Ψ34, *n* = 3; mt-tRNA: Ψ31, *n* = 1; Ψ32, *n* = 3). **c**, Sequence motif of RPUSD1-dependent Ψ sites in human tRNAs. **d**, Sequence motif of RPUSD2-dependent Ψ sites in human tRNAs. **e**, Scatter plot illustrating the distribution of all Ψ sites across human rRNAs and tRNAs following RPUSD4 KD. Ψ1397 in human 16S mt-rRNA is highlighted to reveal a decrease in stoichiometry upon RPUSD4 KD. **f**, Scatter plot illustrating the distribution of all Ψ sites across human rRNAs and tRNAs following RPUSD3 depletion. Source data

**Extended Data Fig. 6:**
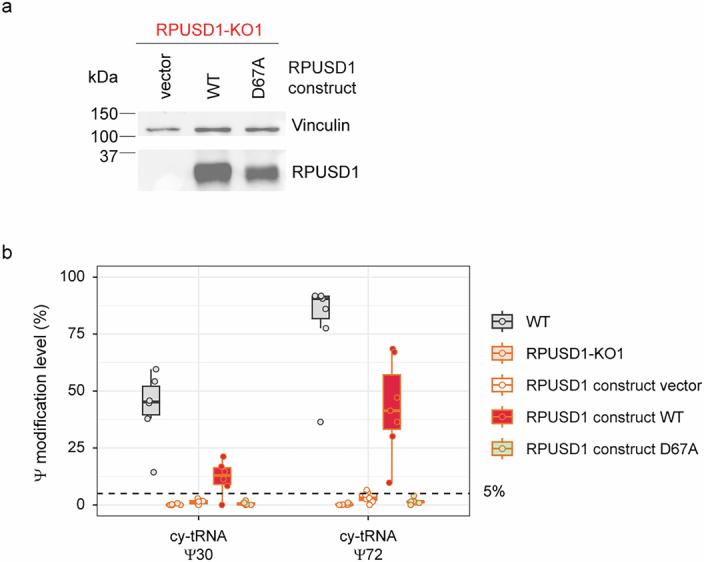
Validation of RPUSD1 enzymatic activities by rescue experiments. **(a)** Representative immunoblots from two independent experiments depict the efficiency of RPUSD1 expression in the RPUSD1-KO1 cells. Anti-Vinculin served as a loading control. **(b)** Comparison of the modification levels of Ψ sites at selected positions of human tRNAs upon RPUSD1 rescue. Box plots visualize all Ψ sites at each position; boxes represent the 25th to 75th percentiles with a line at the median; whiskers correspond to 1.5 times the interquartile range (cy-tRNA: Ψ30, *n* = 6 Ψ sites; Ψ72, *n* = 7 Ψ sites). Source data

We first focused on RPUSD1 and RPUSD2, two PUS with unknown enzymatic activities (Extended Data Fig. 1). Interestingly, we found that, although these two enzymes both work on tRNAs, they have distinct targets and sequence motifs. RPUSD1 catalyses the formation of Ψ30 and Ψ72 in cy-tRNAs, while RPUSD2 installs Ψ modification at position 31–32 in both cy-tRNAs and mt-tRNAs as well as the anticodon position 34 in cy-tRNAs (Fig. 1a,b and Supplementary Table 4). We further demonstrated that RPUSD1 possesses a strict GKΨRCYW (K = G or U; R = A or G; Y = C or U; W = A or U) motif, which may account for its limited targets (Fig. 1c). In comparison, RPUSD2 adopts a much looser VNΨHWNNDNNH (V = A, C or G; N = A, C, G or U; H = A, C or U; W = A or U; D = A, G or U) motif, enabling more diverse catalytic events (Fig. 1d). It is noted that RPUSD2 is the other PUS that works at anticodon positions (Ψ34 in cy-tRNA^Ile-TAT^) in addition to PUS7 (Ψ35 in cy-tRNA^Tyr-GTA^ and Ψ36 in cy-tRNA^Arg-TCT^)^18^, suggesting that their depletion may affect the decoding fidelity of corresponding cy-tRNAs. To validate RPUSD1 enzymatic activities, we performed rescue experiments via re-expression of the WT or catalytically inactive mutant (D67A) form of RPUSD1 in the RPUSD1-knockout (KO) cells (Extended Data Fig. 6 and Supplementary Table 5). As expected, the expression of WT, but not the mutant form of RPUSD1, successfully rescued the formation of Ψ30 and Ψ72 in cy-tRNAs, consistent with our KO results.

In contrast to targeting tRNAs, RPUSD4 has been demonstrated to install Ψ1397 in 16S mt-rRNA^23^. To confirm this, we generated RPUSD4 heterozygous deletion cells showing a substantial reduction in RPUSD4 level^24^, which can therefore be considered RPUSD4-knockdown (KD) cells (Extended Data Fig. 1). Indeed, we observed that only Ψ1397 in 16S mt-rRNA was substantially reduced upon RPUSD4 KD, thereby confirming previous findings (Fig. 1e and Supplementary Table 4). In comparison, RPUSD3 has lost its catalytic aspartate residue and therefore was predicted to be catalytic inactive^4^ (Extended Data Figs. 1 and 5a). Indeed, our data showed that RPUSD3 does not exhibit any noticeable activity on rRNAs and tRNAs, both of which are considered the canonical targets of PUS enzymes (Fig. 1f and Supplementary Table 4). Therefore, we suggest that RPUSD3 might not be a functional active human PUS in vivo.

### TRUB1 and PUS10, but not TRUB2, are redundant for human cy-tRNA Ψ55

**Fig. 2:**
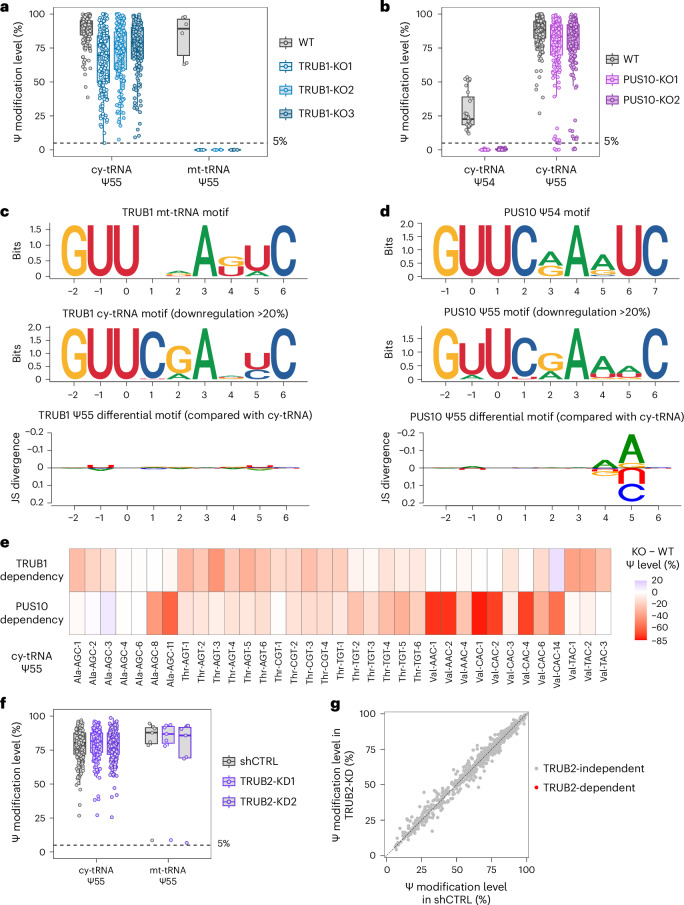
TRUB1 and PUS10 but not TRUB2 are redundant for human cy-tRNA Ψ55. **a**, Comparison of the modification levels of Ψ sites at selected positions of human tRNAs upon TRUB1 depletion. Box plots visualize all Ψ sites at each position; boxes represent the 25th to 75th percentiles with a line at the median; whiskers correspond to 1.5 times the interquartile range (cy-tRNA: Ψ55, *n* = 181 Ψ sites; mt-tRNA: Ψ55, *n* = 6 Ψ sites). Results are adapted from ref. ^18^. **b**, Comparison of the modification levels of Ψ sites at selected positions of human tRNAs upon PUS10 depletion. Box plots visualize all Ψ sites at each position; boxes represent the 25th to 75th percentiles with a line at the median; whiskers correspond to 1.5 times the interquartile range (cy-tRNA: Ψ54, *n* = 28 Ψ sites; Ψ55, *n* = 195 Ψ sites). **c**, Sequence and differential sequence motifs of TRUB1-dependent Ψ sites in human tRNAs. Jensen-Shannon (JS) divergence is used to estimate the difference between motifs. Results are adapted from ref. ^18^. **d**, Sequence and differential sequence motifs of PUS10-dependent Ψ sites in human tRNAs. **e**, Heat map showing the dependency of Ψ55 on TRUB1 and PUS10 across various human tRNA isodecoders. **f**, Comparison of the modification levels of Ψ sites at selected positions of HCT116 tRNAs upon TRUB2 KD. Box plots visualize all Ψ sites at each position; boxes represent the 25th to 75th percentiles with a line at the median; whiskers correspond to 1.5 times the interquartile range (cy-tRNA: Ψ55, *n* = 196 Ψ sites; mt-tRNA: Ψ55, *n* = 7 Ψ sites). **g**, Scatter plot illustrating the distribution of all Ψ sites across HCT116 rRNAs and tRNAs following TRUB2 KD. Source data

In our earlier study, we demonstrated that TRUB1, but not TRUB2, installs all Ψ55 in mt-tRNAs, while depletion of TRUB1 in HeLa cells does not eradicate the Ψ55 in cy-tRNAs, suggesting that there is at least one redundant enzyme responsible for this modification^18^ (Fig. 2a and Supplementary Table 6). TRUB2, another member of the TruB family, was proposed to be involved in the formation of Ψ55 owing to its sequence similarity with TRUB1^25^ (Extended Data Fig. 5b). In addition, PUS10 could serve as another candidate, because its ancestral archaeal Pus10 can catalyse both Ψ54 and Ψ55 in tRNAs^26,27^.

**Extended Data Fig. 7:**
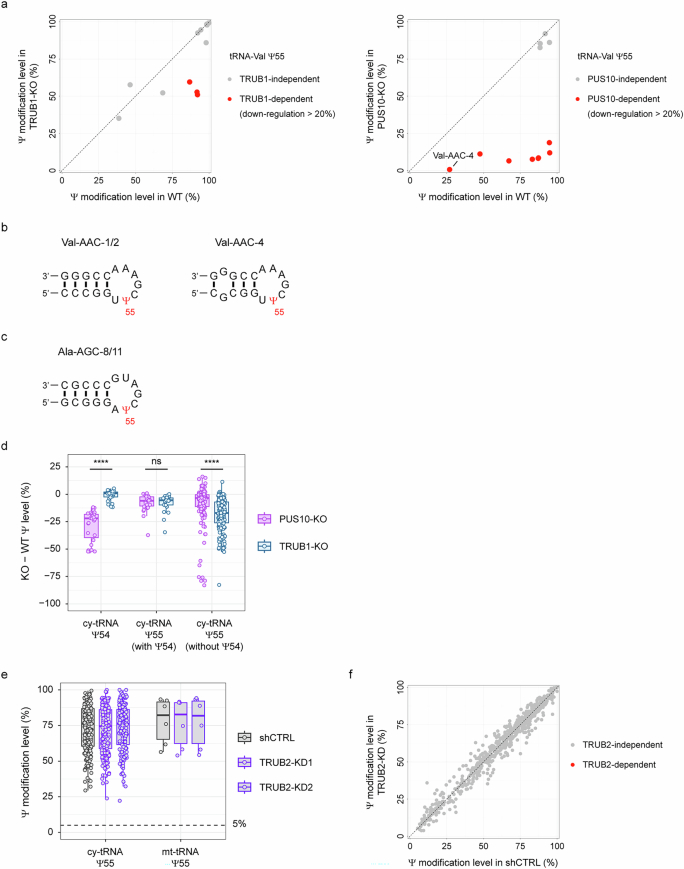
TRUB1 and PUS10 are redundant PUS enzymes responsible for human cy-tRNA Ψ55. **(a)** Scatter plot illustrating all TRUB1-dependent (left panel, in HeLa cells) and PUS10-dependent (right panel, in HCT116 cells) Ψ55 sites in human cy-tRNA^Val^. Each dot represents a cy-tRNA^Val^ isodecoder. **(b)** T-arm structures of human cy-tRNA^Val-AAC-1/2/4^. **(c)** T-arm structures of human cy-tRNA^Ala-AGC-8/11^. **(d)** Comparison of the reduced modification levels of Ψ sites at selected positions in human tRNAs following depletion of PUS10 or TRUB1. Box plots visualize all Ψ sites at each position; boxes represent the 25th to 75th percentiles with a line at the median; whiskers correspond to 1.5 times the interquartile range (cy-tRNA: Ψ54, *n* = 27 Ψ sites; Ψ55 with Ψ54, *n* = 27 Ψ sites; Ψ55 without Ψ54, *n* = 152 Ψ sites). *P* values were calculated using paired, two-tailed *t*-test. ns, *P* ≥ 0.05; *P < 0.05; **P < 0.01; ***P < 0.001 and ****P < 0.0001. **(e)** Comparison of the modification levels of Ψ sites at selected positions of HeLa tRNAs upon TRUB2 knockdown. Box plots visualize all Ψ sites at each position; boxes represent the 25th to 75th percentiles with a line at the median; whiskers correspond to 1.5 times the interquartile range (cy-tRNA: Ψ55, *n* = 183 Ψ sites; mt-tRNA: Ψ55, *n* = 6 Ψ sites). **(f)** Scatter plot illustrating the distribution of all Ψ sites across HeLa rRNAs and tRNAs following TRUB2 knockdown (with depth >=50 in KD samples). Source data

To find the redundant PUS for cy-tRNA Ψ55 modification, we generated PUS10 monoclonal KO in HCT116 cells (Extended Data Fig. 2a,b). Using the PUS10-KO cells, we demonstrated that PUS10 is responsible for both Ψ54 and Ψ55 sites in cy-tRNAs (Fig. 2b and Supplementary Table 4). More specifically, Ψ54 is fully dependent on PUS10, while Ψ55 is only partially dependent, which indicates PUS10 is a redundant enzyme to TRUB1 for cy-tRNA Ψ55. Compared with our earlier results^18^, we found that depletion of TRUB1 can induce a more dramatic decrease of cy-tRNA Ψ55 levels than depletion of PUS10, suggesting that TRUB1 is the major PUS responsible for this modification (Fig. 2a,b). Given the redundancy of TRUB1 and PUS10, we further define their dependent Ψ55 sites as those showing a more than 20% reduction in the corresponding KO cells. Using this criterion, TRUB1-dependent cy-tRNA Ψ55 sites generally possess a GUΨCRANYC (R = A or G; Y = C or U) motif, which varies little from the fundamental tRNA sequence motif (Fig. 2c). By contrast, PUS10 adopts a GΨUCRARUC (R = A or G) motif for Ψ54, while most PUS10-dependent Ψ55 sites share a GUΨCRAWWC (R = A or G; W = A or U) motif, which favours A at the +4 and +5 positions compared with the fundamental tRNA motif (Fig. 2d). Indeed, the differences between TRUB1 and PUS10 motifs can be attributed to their distinct targeting of cy-tRNA subsets, which show varying dependency on these enzymes. However, their activities are not strictly separated, because depletion of neither TRUB1 nor PUS10 can eradicate the Ψ55 site in the vast majority of isodecoders (Fig. 2e). For example, TRUB1 KO reduces Ψ55 levels in cy-tRNA^Val-TAC^ substantially, while PUS10 KO induces greater decrease of Ψ55 levels in cy-tRNA^Val-AAC^ and cy-tRNA^Val-CAC^, but residual Ψ55 modifications are consistently detected in 11 out of 12 isodecoders of cy-tRNA^Val^ in both TRUB1-KO and PUS10-KO cell lines (Extended Data Fig. 7a). The only exception is Ψ55 in cy-tRNA^Val-AAC-4^, which is eliminated after PUS10 depletion. Interestingly, this tRNA isodecoder has a mismatch within its T-stem (Extended Data Fig. 7b). In addition, Ψ55 sites in cy-tRNA^Ala-AGC-8/11^ are also mainly dependent on PUS10, both of which possess GAUCGA within their T-loops (Fig. 2e and Extended Data Fig. 7c). This sequence is not compatible with the TRUB1 GUΨCNA (N = A, C, G or U) motif and therefore is predominantly modified by PUS10. Nevertheless, both TRUB1 KO and PUS10 KO induce only a minor reduction of Ψ55 levels among the cy-tRNAs that possess Ψ54, suggesting that both enzymes can catalyse these Ψ55 modifications efficiently (Extended Data Fig. 7d). Again, this observation confirms that TRUB1 and PUS10 are not strictly separated to certain subsets of cy-tRNAs. Taken together, our results strongly counter the previous model that TRUB1 and PUS10 are not redundant, with their activities restricted to different subsets of cy-tRNAs^28^, but instead indicate that TRUB1 and PUS10 largely function collaboratively. Unlike TRUB1 and PUS10, we were unable to generate a complete TRUB2-KO in HCT116 and HeLa cells, which is consistent with the finding that TRUB2 is essential for a series of human cell lines^29,30^. Alternatively, we generated TRUB2-KD using short hairpin RNA in HCT116 and HeLa cells (Extended Data Fig. 2c). Using BACS, we could not identify any Ψ sites dependent on TRUB2 in both cell lines, suggesting TRUB2 might be another inactive PUS in vivo (Fig. 2f,g, Extended Data Fig. 7e,f and Supplementary Tables 4 and 6).

### The activities of PUS3 and PUSL1 are compartmentalized, acting on tRNAs in distinct cellular locations

**Fig. 3:**
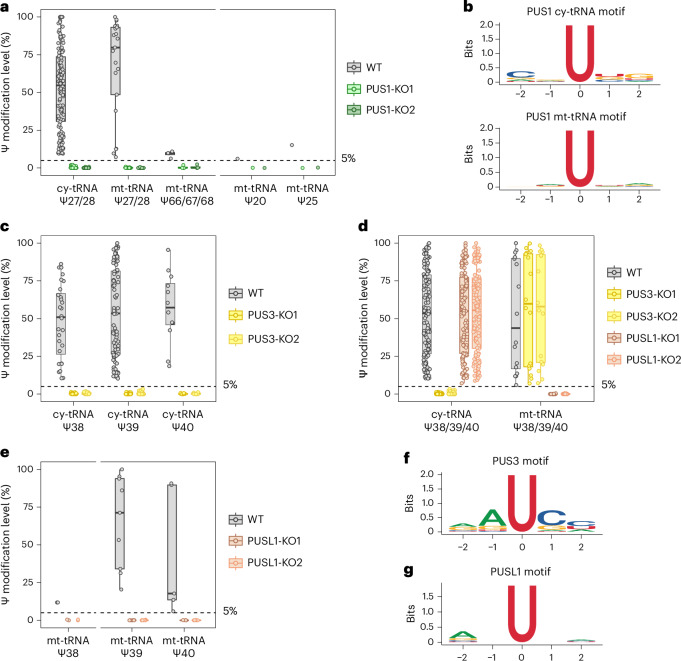
Elucidation of PUS3 and PUSL1 targets in human tRNAs. **a**, Comparison of the modification levels of Ψ sites at selected positions of human tRNAs upon PUS1 depletion. Box plots visualize all Ψ sites at each position; boxes represent the 25th to 75th percentiles with a line at the median; whiskers correspond to 1.5 times the interquartile range (cy-tRNA: Ψ27/28, *n* = 126 Ψ sites; mt-tRNA: Ψ27/28, *n* = 21 Ψ sites; Ψ66/67/68, *n* = 4 Ψ sites; Ψ20, *n* = 1 Ψ site; Ψ25, *n* = 1 Ψ site). Results are adapted from ref. ^18^. **b**, Sequence motifs of PUS1-dependent Ψ sites in human cy-tRNAs (top) and mt-tRNAs (bottom). Results are adapted from ref. ^18^. **c**, Comparison of the modification levels of Ψ sites at selected positions of human tRNAs upon PUS3 depletion. Box plots visualize all Ψ sites at each position; boxes represent the 25th to 75th percentiles with a line at the median; whiskers correspond to 1.5 times the interquartile range (cy-tRNA: Ψ38, *n* = 28 Ψ sites; Ψ39, *n* = 86 Ψ sites; Ψ40, *n* = 12 Ψ sites). **d**, Comparison of Ψ38–40 modification levels in human cy-tRNAs and mt-tRNAs following PUS3 or PUSL1 depletion. Box plots visualize all Ψ sites at each position; boxes represent the 25th to 75th percentiles with a line at the median; whiskers correspond to 1.5 times the interquartile range (cy-tRNA: Ψ38/39/40, *n* = 126 Ψ sites; mt-tRNA: Ψ38/39/40, *n* = 16 Ψ sites). **e**, Comparison of the modification levels of Ψ sites at selected positions of human tRNAs upon PUSL1 depletion. Box plots visualize all Ψ sites at each position; boxes represent the 25th to 75th percentiles with a line at the median; whiskers correspond to 1.5 times the interquartile range (mt-tRNA: Ψ38, *n* = 2 Ψ sites; Ψ39, *n* = 9 Ψ sites; Ψ40, *n* = 5 Ψ sites). **f**, Sequence motif of PUS3-dependent Ψ sites in human tRNAs. **g**, Sequence motif of PUSL1-dependent Ψ sites in human tRNAs. Source data

PUS1, PUS3 and PUSL1 are the three human PUS that belong to the TruA family. Our earlier study has shown that PUS1 is predominantly responsible for Ψ27–28 in both cy-tRNAs and mt-tRNAs^18^ (Fig. 3a and Supplementary Table 6). Although there are only a few studies on the enzymatic activities of human PUS3, it is predicted to modify Ψ38–40 in both cy-tRNAs and mt-tRNAs^4^, similar to PUS1. Recent research confirms PUS3 can modify Ψ39 in human cy-tRNAs^31,32^. To further elucidate PUS3 activities, we quantified the Ψ levels in PUS3-KO cells using BACS (Extended Data Fig. 3). Surprisingly, we found that PUS3 activities are restricted to Ψ38–40 in cy-tRNAs, indicating there is another enzyme catalysing the Ψ38–40 modification in mt-tRNAs (Fig. 3c,d and Supplementary Table 4). Given that PUSL1 has been identified as a PUS that predominantly localized to mitochondria^33^, we proposed that it would be the responsible PUS (Extended Data Fig. 3). Indeed, PUSL1 KO leads to a depletion of Ψ38–40 in mt-tRNAs (Fig. 3d,e and Supplementary Table 4). More specifically, PUSL1 can modify Ψ39 in mt-tRNA^Phe^, which can serve as structural constituents in the mitoribosomal large subunit (mt-LSU). Taken together, the enzymatic activities of PUSL1 on mt-tRNAs may provide a possible explanation for why PUSL1 is required for efficient mitochondrial translation^33^. We further analysed the sequence motifs of PUS3 and PUSL1. Similar to PUS1^18^, both PUS3 and PUSL1 do not possess a clear sequence motif, suggesting that they may also rely on structural motifs (Fig. 3b,f,g). Our motif analysis aligns with earlier reports on PUS1^16^ and PUS3^32^, suggesting this substrate preference may be conserved across TruA family enzymes.

### PUS7L displays unique catalytic activities within the variable loops

**Fig. 4:**
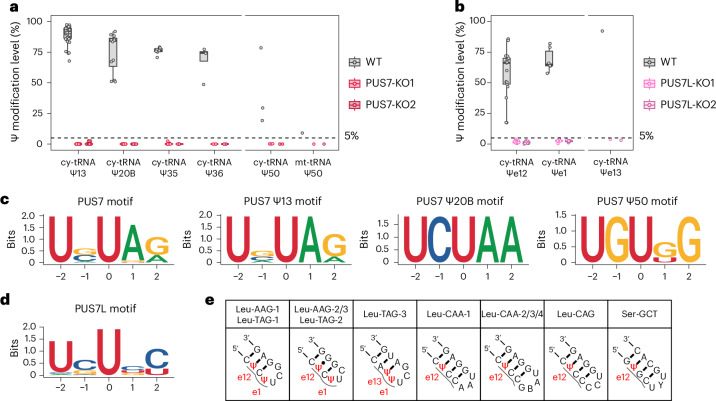
Human PUS7L catalyses pseudouridylation within the variable loops of cy-tRNAs. **a**, Comparison of the modification levels of Ψ sites at selected positions of human tRNAs upon PUS7 depletion. Box plots visualize all Ψ sites at each position; boxes represent the 25th to 75th percentiles with a line at the median; whiskers correspond to 1.5 times the interquartile range (cy-tRNA: Ψ13, *n* = 43 Ψ sites; Ψ20B, *n* = 12 Ψ sites; Ψ35, *n* = 7 Ψ sites; Ψ36, *n* = 4 Ψ sites; Ψ50, *n* = 3 Ψ sites; mt-tRNA: Ψ50, *n* = 1 Ψ site). Results are adapted from ref. ^18^. **b**, Comparison of the modification levels of Ψ sites at selected positions of human tRNAs upon PUS7L depletion. Box plots visualize all Ψ sites at each position; boxes represent the 25th to 75th percentiles with a line at the median; whiskers correspond to 1.5 times the interquartile range (cy-tRNA: Ψe12, *n* = 17 Ψ sites; Ψe1, *n* = 6 Ψ sites; Ψe13, *n* = 1 Ψ site). **c**, Sequence motif of PUS7-dependent Ψ sites in human tRNAs. Results are adapted from ref. ^18^. **d**, Sequence motif of PUS7L-dependent Ψ sites in human tRNAs. **e**, Variable loop structures of PUS7L targets. Source data

PUS7L, a homologue to PUS7, has long been a mysterious PUS enzyme since its discovery. Previously, we have revealed PUS7 modifies 5 and 1 positions in cy-tRNAs and mt-tRNAs, respectively^18^ (Fig. 4a and Supplementary Table 6). To understand PUS7L enzymatic activities, we profiled the Ψ landscape of PUS7L-KO cells (Extended Data Fig. 4). Interestingly, we observed a clear reduction in modification levels of Ψe12 and Ψe1 sites, both of which are located within the variable loops (Fig. 4b and Supplementary Table 4). We further analysed its sequence preference within cy-tRNAs. In stark contrast to the UNΨAR (N = A, C, G or U; R = A or G) plus UGΨKG (K = G or U) motif for PUS7^18^, we could identify only a weak UNΨBY (N = A, C, G or U; B = C, G or U; Y = C or U) motif for PUS7L (Fig. 4c,d). Notably, PUS7L also displays a relatively high activity in a unique isodecoder cy-tRNA^Leu-TAG-3^, where it modifies the e13 and e1 positions (Fig. 4e). Given the close proximity of e12, e13 and e1 positions, we proposed that PUS7L may rely on a structural motif to modify Ψ sites within the 5′-side of variable loops, in a way that is similar to the TruA family members PUS1, PUS3 and PUSL1. We therefore plotted the structures of variable loops in cy-tRNA^Leu^ and cy-tRNA^Ser^ (Fig. 4e). Indeed, only uridines located within the e12, e13 and unpaired e1 positions will be modified by PUS7L. The detailed mechanism underlying the distinct enzymatic properties of PUS7L compared with PUS7 requires further investigation.

### A comprehensive PUS-dependent Ψ map of human tRNAs

**Fig. 5:**
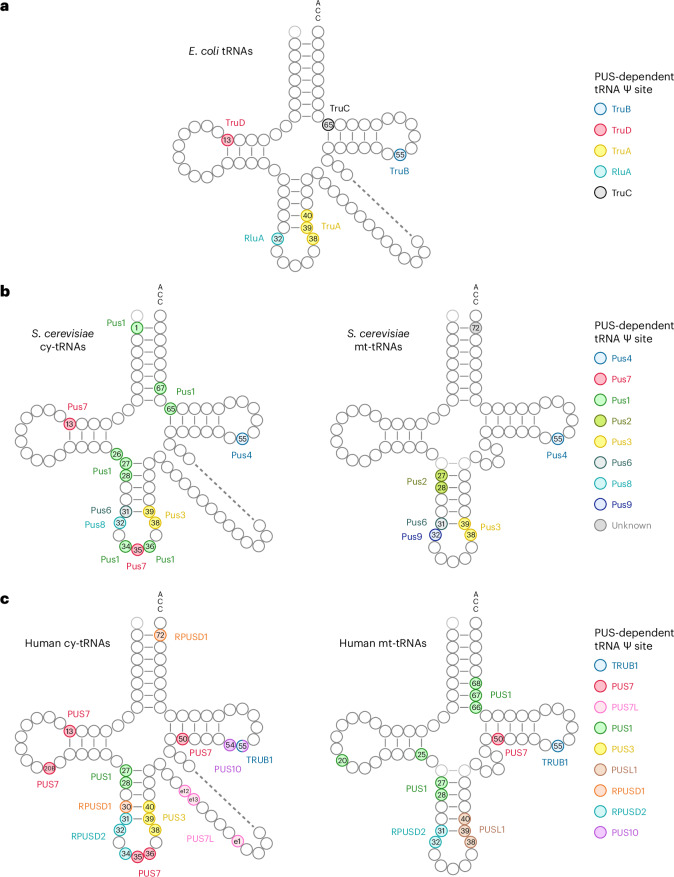
Comprehensive PUS-dependent Ψ landscape of tRNAs. **a**–**c**, Integrated view of the PUS-dependent Ψ profiles of *E. coli* (**a**), *S. cerevisiae* (**b**) and human (**c**) tRNAs. Results for *E. coli* and *S. cerevisiae* are adapted from ref. ^35^.

**Extended Data Fig. 8:**
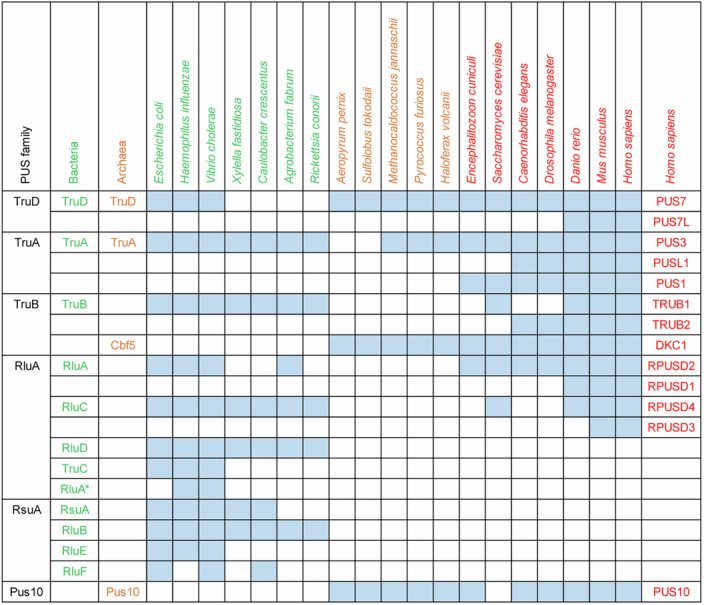
Distribution of PUS enzymes across various species. PUS enzymes are aligned based on their sequence similarities and enzymatic activities. Green, orange, and red color denote bacteria, archaea, and eukarya, respectively. *An uncharacterized PUS within the RluA family (NCBI TIGR01621). Source data

By integrating the above results with our earlier data on TRUB1, PUS7 and PUS1^18^, we can generate a comprehensive PUS-dependent Ψ map of human tRNAs (Fig. 5, Extended Data Fig. 8 and Supplementary Fig. 1). Compared with the previously established *E. coli* and *S. cerevisiae* maps^34–36^, human cells possess many more abundant Ψ sites within tRNAs. Consistent with this, there are more PUS enzymes acting on tRNAs in human cells compared with bacteria and yeast. Even conserved PUS enzymes exhibit broader activity compared with their bacterial and yeast homologues. Together, these observations suggest a more important role for Ψ in the biogenesis of human tRNA.

### Human PUS enzymes modify pre-tRNAs at different stages

In eukaryotes, nuclear tRNA genes are initially transcribed as pre-tRNAs, which will undergo a series of processing steps, including removal of the 5′-leader and 3′-trailer sequences, addition of CCA to the 3′-end, splicing of the intron if presents, and introducing a wide range of chemical modifications^37,38^. Given the high abundance of Ψ in mature cy-tRNAs, understanding the temporal order of pseudouridylation could provide valuable insights into the pre-tRNA processing. However, the Ψ profile of pre-tRNAs is only partially elucidated in yeast^39–41^ and *Xenopus laevis*^42^. With BACS and the comprehensive PUS-dependent Ψ map of human tRNAs, we were able to investigate the specific stage of pre-tRNA processing at which each PUS enzyme introduces Ψ modifications. To detect Ψ modifications in human pre-tRNAs, we applied stringent filtering to the BACS reads generated from HeLa cells. More specifically, we discarded all reads that were mapped to mature cy-tRNAs but kept the reads covering at least one of the 5′-leader, 3′-trailer or intron sequences of pre-tRNAs.

**Fig. 6:**
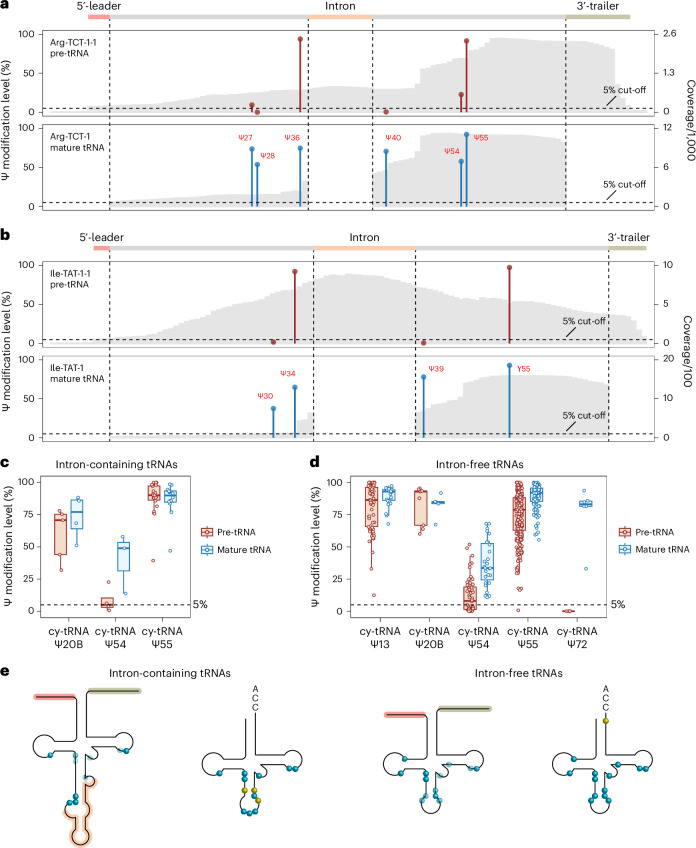
Investigation of Ψ modification in human pre-tRNAs. **a**, Comparison of Ψ modification levels between human pre-tRNA^Arg-TCT-1-1^ and mature cy-tRNA^Arg-TCT-1^. **b**, Comparison of Ψ modification levels between human pre-tRNA^Ile-TAT-1-1^ and mature cy-tRNA^Ile-TAT-1^. **c**, Comparison of Ψ modification levels at selected positions in human intron-containing pre-tRNAs and mature tRNAs. Box plots visualize all Ψ sites at each position; boxes represent the 25th to 75th percentiles with a line at the median; whiskers correspond to 1.5 times the interquartile range (pre-tRNA: Ψ20B, *n* = 5 Ψ sites; Ψ54, *n* = 4 Ψ sites; Ψ55, *n* = 22 Ψ sites; mature tRNA: Ψ20B, *n* = 4 Ψ sites; Ψ54, *n* = 3 Ψ sites; Ψ55, *n* = 16 Ψ sites). **d**, Comparison of Ψ modification levels at selected positions in human intron-free pre-tRNAs and mature tRNAs. Box plots visualize all Ψ sites at each position; boxes represent the 25th to 75th percentiles with a line at the median; whiskers correspond to 1.5 times the interquartile range (pre-tRNA: Ψ13, *n* = 63 Ψ sites, Ψ20B, *n* = 9 Ψ sites; Ψ54, *n* = 52 Ψ sites; Ψ55, *n* = 261 Ψ sites; Ψ72, *n* = 6 Ψ sites; mature tRNA: Ψ13, *n* = 32 Ψ sites, Ψ20B, *n* = 5 Ψ sites; Ψ54, *n* = 27 Ψ sites; Ψ55, *n* = 143 Ψ sites; Ψ72, *n* = 6 Ψ sites). **e**, Summary of Ψ profiles in human pre-tRNAs and mature tRNAs. The 5′-leader, intron and 3′-trailer sequences are highlighted in red, orange and green, respectively. Blue dots indicate Ψ sites present at the pre-tRNA stage, while yellow dots denote Ψ sites specific to the mature tRNA stage. Colour shading reflects relative Ψ modification levels. Source data

**Extended Data Fig. 9:**
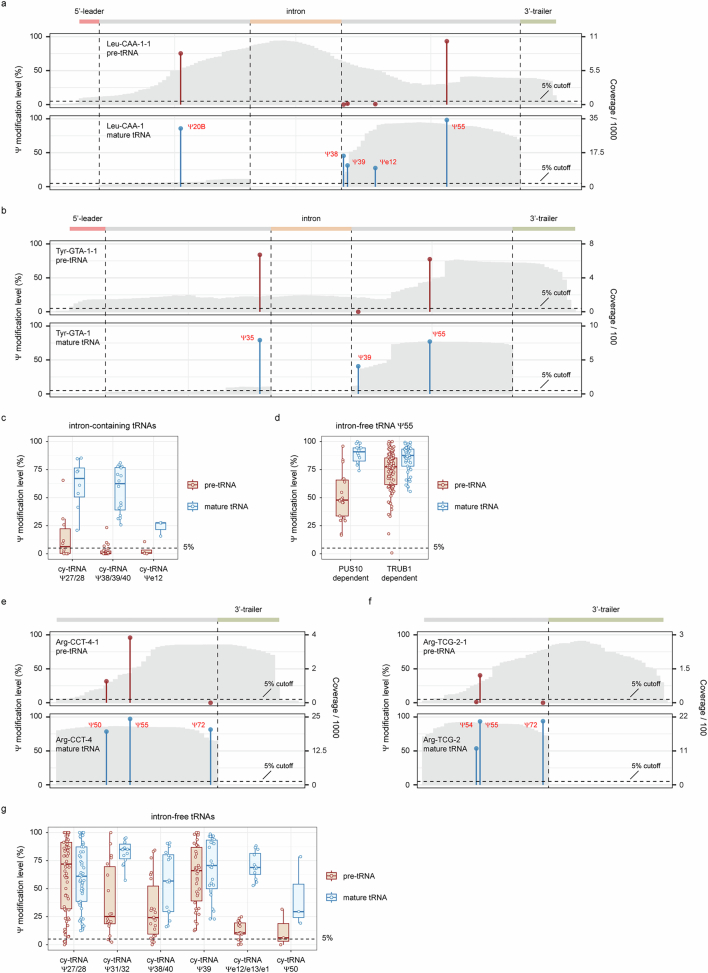
Mapping of Ψ in human pre-tRNAs. **(a)** Comparison of Ψ modification levels between human pre-tRNA^Leu-CAA-1-1^ and mature cy-tRNA^Leu-CAA-1^. **(b)** Comparison of Ψ modification levels between human pre-tRNA^Tyr-GTA-1-1^ and mature cy-tRNA^Tyr-GTA-1^. **(c)** Comparison of Ψ modification levels at selected positions in human intron-containing pre-tRNAs and mature tRNAs. Box plots visualize all Ψ sites at each position; boxes represent the 25th to 75th percentiles with a line at the median; whiskers correspond to 1.5 times the interquartile range (pre-tRNA: Ψ27/28, *n* = 10 Ψ sites; Ψ38/39/40, *n* = 22 Ψ sites; Ψe12, *n* = 4 Ψ sites; mature tRNA: Ψ27/28, *n* = 8 Ψ sites; Ψ38/39/40, *n* = 16 Ψ sites; Ψe12, *n* = 3 Ψ sites). **(d)** Comparison of PUS10- and TRUB1-dependent Ψ55 modification levels in human intron-free pre-tRNAs and mature tRNAs. Box plots visualize all Ψ sites at each position; boxes represent the 25th to 75th percentiles with a line at the median; whiskers correspond to 1.5 times the interquartile range (PUS10-dependent: pre-tRNA, *n* = 21 Ψ sites; mature tRNA, *n* = 14 Ψ sites; TRUB1-dependent: pre-tRNA, *n* = 97 Ψ sites; mature tRNA, *n* = 49 Ψ sites). **(e)** Comparison of Ψ modification levels between human pre-tRNA^Arg-CCT-4-1^ and mature cy-tRNA^Arg-CTT-4^. **(f)** Comparison of Ψ modification levels between human pre-tRNA^Arg-TCG-2-1^ and mature cy-tRNA^Arg-TCG-2^. **(g)** Comparison of Ψ modification levels at selected positions in human intron-free pre-tRNAs and mature tRNAs. Box plots visualize all Ψ sites at each position; boxes represent the 25th to 75th percentiles with a line at the median; whiskers correspond to 1.5 times the interquartile range (pre-tRNA: Ψ27/28, *n* = 69 Ψ sites; Ψ31/32, *n* = 18 Ψ sites; Ψ38/40, *n* = 22 Ψ sites; Ψ39, *n* = 38 Ψ sites; Ψe12/e13/e1, *n* = 18 Ψ sites; Ψ50, *n* = 3 Ψ sites; mature tRNA: Ψ27/28, *n* = 49 Ψ sites; Ψ31/32, *n* = 14 Ψ sites; Ψ38/40, *n* = 16 Ψ sites; Ψ39, *n* = 26 Ψ sites; Ψe12/e13/e1, *n* = 12 Ψ sites; Ψ50, *n* = 3 Ψ sites).

We first focused on intron-containing pre-tRNAs in human cells, including pre-tRNA^Arg-TCT^, pre-tRNA^Ile-TAT^, pre-tRNA^Leu-CAA^ and pre-tRNA^Tyr-GTA^. Interestingly, we found these pre-tRNAs possess several intron-sensitive Ψ sites, especially for those localized close to introns, such as RPUSD1-dependent Ψ30 and PUS3-dependent Ψ38–40 (Fig. 6a,b and Extended Data Fig. 9a–c). Interestingly, the intron of pre-tRNA^Leu-CAA^ also largely blocks the formation of PUS7L-dependent Ψe12, which is relatively far from the intron (Extended Data Fig. 9a,c). In addition, Ψ27–28 sites are also partially modified by PUS1 in pre-tRNA^Arg-TCT^ (Extended Data Fig. 9c). By contrast, the anticodon Ψ34/35/36 sites are all introduced before the intron splicing, which is consistent with their yeast counterparts^43,44^ (Fig. 6a,b and Extended Data Fig. 9b). It is noted that, although all three anticodon positions can be modified in yeast and human cells, different enzymes are responsible for these modification events. Ψ34 and Ψ36 are both modified by Pus1p in yeast cy-tRNA^Ile-TAT^ (ref. ^45^), while human RPUSD2 and PUS7 modify Ψ34 in cy-tRNA^Ile-TAT^ and Ψ36 in cy-tRNA^Arg-TCT^, respectively. Only Ψ35 in cy-tRNA^Tyr-GTA^ is conserved installed by yeast Pus7p^12^ and its human homologue PUS7. Despite being catalysed by different PUS enzymes, Ψ modifications within anticodons in human and yeast cells are introduced in a similar manner, indicating the importance of tRNA introns. In addition to the above Ψ sites, we also observed that pre-tRNA^Leu-CAA^ Ψ20B and pre-tRNA^Arg-TCT^ Ψ54/55 are introduced before the trimming of 5′-leader and 3′-trailer sequences, respectively, suggesting these Ψ sites are installed at a very early stage of pre-tRNA processing (Fig. 6a,c and Extended Data Fig. 9a).

We further expanded our analysis to intron-free pre-tRNAs, which represent the majority of pre-tRNAs in human cells. Consistent with intron-containing pre-tRNAs, we confirmed PUS7-dependent Ψ13 and Ψ20B sites and TRUB1-dependent Ψ55 sites are highly modified before the trimming of the 5′-leader and 3′-trailer sequences of intron-free pre-tRNAs, respectively (Fig. 6d and Extended Data Fig. 9d). However, PUS10-dependent Ψ55 sites are not fully modified before the removal of the 3′-trailer sequence (Extended Data Fig. 9d). Interestingly, Ψ54, another PUS10-dependent site, is also partially modified at the same stage (Fig. 6d). By contrast, Ψ72, a site present only in cy-tRNA^Arg-CCT^ and cy-tRNA^Arg-TCG^, is not found in their pre-tRNAs (Fig. 6d and Extended Data Fig. 9e,f). This phenomenon could be explained by the altered secondary structure and the absence of a 3′-CCA end. Notably, 5-methylcytidine (m^5^C) at position 72 is also found to be installed in the mature tRNA stage^46^, where the 3′-CCA end plays an important role for NSUN6 recognition^47^, suggesting a similar mechanism for RPUSD1 recognition of its Ψ72 substrate. Due to short read lengths and the difficulty in mapping, we had low coverage of Ψ sites located in the middle of intron-free tRNAs, including Ψ27/28, Ψ30/31/32, Ψ38/39/40, Ψe12/e13/e1 and Ψ50. Nevertheless, our results suggest that PUS1-dependent Ψ27/28 and PUS3-dependent Ψ39 display comparable modification levels in pre-tRNAs and mature cy-tRNAs, while Ψ38/40 have lower stoichiometries in pre-tRNAs compared with mature cy-tRNAs (Extended Data Fig. 9g). Similarly, RPUSD2-dependent Ψ31/32, PUS7L-dependent Ψe12/e13/e1 and PUS7-dependent Ψ50 can all be installed at the pre-tRNA stage, but their stoichiometries are substantially lower than the corresponding sites in mature cy-tRNAs (Extended Data Fig. 9g). We were unable to map Ψ30 in intron-free pre-tRNAs owing to insufficient coverage, and its modification status requires further investigation. Taken together, we demonstrated that PUS enzymes modify Ψ at different stages of pre-tRNA processing (Fig. 6e).

## Discussion

Ψ has long been an enigmatic RNA modification, despite its high abundance in human cellular RNAs. This issue can be attributed to the highly sophisticated landscape of Ψ in human cells compared with those of bacteria and yeast and the lack of ideal Ψ sequencing methods. To overcome this problem, we used BACS to map the Ψ profile of 12 individual PUS-KO or PUS-KD cell lines and elucidated the primary targets of all stand-alone PUS enzymes in human cells, including PUS1, PUS3, PUSL1, PUS7, PUS7L, RPUSD1–4, TRUB1, TRUB2 and PUS10. Our results demonstrated a conserved but also much more sophisticated PUS-dependent Ψ map of human tRNAs compared with *E. coli* and *S. cerevisiae*. Benefitting from this comprehensive map, we further revealed the temporal order of pseudouridylation in pre-tRNA processing by different human PUS enzymes.

PUS can be divided into six different families, named after the bacterial or archaeal enzymes: TruA, TruB, TruD, RluA, RsuA and Pus10. Human PUS enzymes cover all except the RsuA family (Supplementary Fig. 1). Among them, the TruD family has been proposed as the most ancient one^34^. In most species, there is only one conserved member within this family, named TruD for bacteria and archaea, and PUS7 for eukaryotes (Extended Data Fig. 8). Nevertheless, we observed substantial expansion of their activities during evolution. In bacteria, archaea or lower eukaryotes such as yeast, TruD/Pus7p is responsible for only one or two Ψ sites (Ψ13 or Ψ13 and Ψ35), while human PUS7 can catalyse three additional Ψ sites, including Ψ20B, Ψ36 and Ψ50, suggesting that it may play a more important role in tRNA modification. In addition to the conserved PUS7, vertebrates also evolved another enzyme belonging to the TruD family, namely PUS7L (Extended Data Fig. 8), which exhibits unique enzymatic activities towards e12, e13 and e1 positions within the variable loops. Compared with PUS7, PUS7L lacks a well-defined sequence motif and instead functions in a manner that induces consecutive Ψ sites, resembling the activity of TruA family members. This difference indicates that even PUS enzymes within the same family may still display distinct properties in pseudouridylation, highlighting the importance of having a comprehensive PUS-dependent Ψ map.

Within the TruA family, *E. coli* TruA, *S. cerevisiae* Pus3p and human PUS3 appear to be well-conserved PUS enzymes (Extended Data Fig. 8), as all of them catalyse the formation of Ψ38–40 within the anticodon arm, suggesting the importance of these Ψ modifications. Human and other higher eukaryotes also evolved a unique PUS3 homologue, PUSL1, to catalyse the pseudouridylation of Ψ38–40 in mt-tRNAs (Extended Data Fig. 8). In addition to PUS3 and PUSL1, PUS1 is another TruA family member found in most eukaryotes, from yeast to humans (Extended Data Fig. 8). PUS1 shares similarity with PUS3 and PUSL1 in that they mostly rely on structural motifs to modify consecutive Ψ sites. However, in contrast to PUS3, PUS1 mainly targets Ψ27–28 within the anticodon stem. Although PUS1 and PUS3 can both induce consecutive Ψ sites, PUS1 clearly shows more diverse activities, exemplified by Ψ1, Ψ34, Ψ36, Ψ65 and Ψ67 in yeast cy-tRNAs, and Ψ20, Ψ25 and Ψ66–68 in human mt-tRNAs. In human cells, PUS1 appears to be a major PUS enzyme in mitochondria, as we previously demonstrated PUS1 is also responsible for all eight Ψ sites within mt-mRNAs^18^.

Three human PUS enzymes belong to the TruB family, including two stand-alone enzymes, TRUB1 and TRUB2, as well as one snoRNA-dependent enzyme, DKC1 (Extended Data Fig. 8). DKC1 relies on box H/ACA snoRNA to guide site-specific pseudouridylation, which predominantly works on cytosolic rRNAs and spliceosomal snRNAs. In comparison, stand-alone TRUB1 and TRUB2 are traditionally believed to act on tRNA position 55, as their bacterial and yeast homologues^4^. The distinct PUS10 family, present in archaea and humans but absent in bacteria and yeast, exhibits similar enzymatic activity at position 55, along with additional activity at position 54 (Extended Data Fig. 8). Therefore, TRUB1, TRUB2 and PUS10 are predicted to be potential redundant PUS enzymes for Ψ55 in cy-tRNAs and mt-tRNAs^4^. However, our results strongly indicate that only TRUB1 and PUS10 are functional active enzymes responsible for Ψ55 in cy-tRNAs, and TRUB1 also serves as the sole responsible PUS enzyme for Ψ55 in mt-tRNAs. By contrast, TRUB2 does not display noticeable PUS enzymatic activities in both cy-tRNAs and mt-tRNAs. Interestingly, earlier screening indicated that TRUB2 is directly involved in the mitochondrial PUS module together with RPUSD4 and RPUSD3, and the biochemical study of TRUB2 suggested its possible role in mt-LSU assembly^48^. However, the catalytic domain of TRUB2 has diverged from that of other members within the TruB family, which may account for its loss of catalytic activity in vivo (Extended Data Fig. 5b). Moreover, TRUB2 is predicted to form a complex with MTERF3^49^, a highly conserved member in the MTERF family that plays critical roles in mitochondrial ribosomal biogenesis^50^ (Extended Data Fig. 5c). It is noted that another member within this family, MTERF4, forms a complex with NSUN4, the 12S mt-rRNA m^5^C methyltransferase^51^. The NSUN4–MTERF4 complex has also been proposed to play an essential role in mt-LSU assembly^52^, which has recently been confirmed by cryo-electron microscopy^53–56^. Compared with its bacterial counterparts RsmB^57^ and RsmF^58,59^, NSUN4 has lost its RNA-binding domain during evolution^60^. Therefore, it needs to bind MTERF4 before acting on mt-rRNAs. We suggest that a similar scenario may apply to TRUB2, as DKC1—another member of the TruB family—retains both the PUS domain and the archaeosine transglycosylase (PUA) domain, which enables its interaction with box H/ACA snoRNA^61^ (Extended Data Fig. 5c). Mutation in the catalytic domain, loss of the PUA domain and binding with MTERF3 may relocate TRUB2 function from mt-tRNA pseudouridylation to mitoribosome assembly. Aligned with this view, TRUB2 appears to be well conserved across the metazoan, consistent with its functional importance (Extended Data Fig. 8). Conversely, TRUB1 is predicted to be lost in *Caenorhabditis* *elegans* and *Drosophila*, as its enzymatic functions can be compensated by PUS10 (Extended Data Fig. 8). This functional redundancy is also believed to account for the loss of PUS10 in *S. cerevisiae* and other species^62^. In human cells, we found that TRUB1 and PUS10 largely work in a collaborative rather than separated manner, although both of them display preference towards certain subsets of cy-tRNAs.

RluA family is one of the most sophisticated PUS families, due to its dual activities on tRNAs and rRNAs (Supplementary Fig. 1). *E. coli* possesses four members of RluA family: RluA, RluC, RluD and TruC^34^. Among them, RluA works on both tRNAs and rRNAs, while TruC and RluC/RluD specifically target tRNAs and rRNAs, respectively (Supplementary Fig. 1). It is noted that RluA family has not been identified in archaea, suggesting eukaryotes inherited this family solely from bacteria (Extended Data Fig. 8). Indeed, bacterial RluA and RluC may serve as the ancestors of eukaryotic RPUSD2 and RPUSD4, respectively (Extended Data Fig. 8). Compared with bacterial RluA, eukaryotic RPUSD2 has lost its activity towards rRNAs, while its function on tRNA Ψ32 is well maintained. Yeast evolved three RluA homologues, namely Pus6p, Pus8p and Pus9p, and expanded their activities to modify adjacent tRNA Ψ31 and Ψ32. In human cells, RPUSD2 itself can catalyse the Ψ31 and Ψ32 modifications in both cy-tRNAs and mt-tRNAs as well as Ψ34 within the anticodon. Vertebrates further evolved RPUSD1, a close homologue to RPUSD2 (Extended Data Fig. 8), to catalyse cy-tRNA Ψ30 and Ψ72. However, RPUSD1 works only on cy-tRNAs and displays distinct sequence preferences from RPUSD2, which may suggest their functional divergence. In contrast to the tRNA enzymes RPUSD1/2, RPUSD4 is well conserved from bacterial RluC (Extended Data Fig. 8), as all consistently catalyse a highly conserved Ψ site in bacterial or mitochondrial 16S rRNA, suggesting their biological importance. However, we noticed that some species, such as *C. elegans* and *Drosophila*, may have lost their RPUSD4 homologues during evolution (Extended Data Fig. 8). In vertebrates, there is a close homologue to RPUSD4, named RPUSD3, yet with its catalytic aspartate residue mutated to glycine (Extended Data Fig. 5a and 8). Consistent with this, we confirmed that RPUSD3 might not be a functional active PUS enzyme in vivo. The actual biological function of RPUSD3 remains to be discovered.

Despite the divergence of stand-alone PUS activities among different species, their Ψ targets are primarily determined by two factors: the subcellular localization of the PUS enzyme and its preferred Ψ-recognition motifs. Proper localization is the prerequisite for PUS to modify certain RNAs. For example, human PUSL1^33^ and RPUSD4^23,24^ predominantly localize to the mitochondria, and their activities are restricted to mt-tRNAs and 16S mt-rRNAs, respectively. Certain enzymes, such as PUS1^15,20^ and TRUB1^7^, are localized to both the nucleus and mitochondria, which may explain their ability to modify both cy-tRNAs and mt-tRNAs. Beyond localization, most stand-alone PUS preferentially target certain secondary or tertiary structures in tRNAs (Supplementary Fig. 2). For instance, RPUSD2 mainly targets the first unpaired uridine within the anticodon loop, while PUS1/PUS3/PUSL1 and PUS7L can modify multiple consecutive uridines within the anticodon arm and the variable loop, respectively. In addition, some stand-alone PUS enzymes recognize specific sequence motifs for pseudouridylation, such as the GUΨNNA (N = A, C, G or U) motif within the stem–loop structures for TRUB1 and the UNΨAR (N = A, C, G or U; R = A or G) motif within more variable structures for PUS7.

The above theory could explain why different PUS are utilized to modify the same Ψ sites among different species. For example, both Ψ34 and Ψ36 are induced by Pus1p in yeast pre-tRNA^Ile-TAT^, while human pre-tRNA^Ile-TAT^ possesses only RPUSD2-dependent Ψ34. This divergence could be attributed to the different intron sequences and different secondary structures of introns between yeast and human pre-tRNA^Ile-TAT^ (Supplementary Fig. 3). Yeast pre-tRNA^Ile-TAT^ contains a much longer intron sequence that is proposed to fold into a structure that favours yeast Pus1p for Ψ34 and Ψ36 modifications, because the local structure is similar to that of its canonical substrates Ψ27/28. In comparison, human pre-tRNA^Ile-TAT^ intron is proposed to fold in a different way, with U34 located in a stem–loop structure that is analogous to an extended anticodon arm, which is more suitable for RPUSD2 modification. Because RPUSD2 modifies only the first unpaired uridine in the anticodon loop, U36 is not modified in human pre-tRNA^Ile-TAT^. Similarly, the human pre-tRNA^Arg-TCT^ anticodon loop possesses a UCUAG motif, where U36 can serve as an ideal substrate for PUS7 modification.

Ψ and human PUS enzymes have recently garnered increasing attention, with emerging evidence linking human PUS enzymes to genetic diseases and cancer^3^. However, most research on human stand-alone PUS has been focusing on those conserved from their yeast homologyes, such as PUS1^15,63^, PUS3^31,32,64^ and PUS7^10,11,65–67^. The difficulty in identifying the true targets of human PUS hampers their further investigation. Here we mainly focused on the canonical targets of 12 human stand-alone PUS, especially tRNAs. We uncovered that human stand-alone PUS enzymes are generally involved in more diverse Ψ modification events than their yeast homologues, indicating there is a clear need to revisit earlier human PUS research that relied on yeast results. In addition, our PUS-dependent Ψ map provides a valuable resource for studying PUS enzymes in other key model organisms, including *C. elegans*, *Drosophila* and zebrafish. We anticipate that our BACS method and comprehensive PUS-dependent tRNA Ψ map can be widely adopted to elucidate the biological functions of Ψ and human PUS enzymes.

## Methods

### Cell culture

HCT116 (American Type Culture Collection (ATCC), CCL-247) and HeLa (gifted from P. J. Ratcliffe, University of Oxford; originally obtained from ATCC, CCL-2) cells were cultured in McCoy’s 5A (Modified) Medium (Gibco) and Dulbecco’s modified Eagle medium (Gibco), respectively, supplemented with 10% (v/v) foetal bovine serum (Gibco) and 1% penicillin–streptomycin (Gibco) at 37 °C with 5% CO_2_. The HCT116 cell line was authenticated by ATCC short tandem repeat (STR) profiling.

### Generation of CRISPR KO cell lines

Monoclonal PUS-KO HCT116 cells were generated using CRISPR–Cas9 technology. In brief, single guide RNA sequences were cloned into PX459 plasmids^68^. Transfection was performed using Lipofectamine 3000 Transfection Reagent (Invitrogen) following the manufacturer’s protocol. Cells were then selected by 2 µg ml^−1^ puromycin (Thermo). Serial dilution was performed to achieve clonal isolation. Finally, clones were expanded and picked for western blot and Sanger sequencing validation. The single guide RNA sequences are presented in Supplementary Table 2.

### KD of TRUB2 in HCT116 and HeLa cells

Lentiviral vectors carrying short hairpin RNAs targeting TRUB2 (Supplementary Table 2, Sigma) were packaged using calcium chloride (Sigma) to produce viral supernatant, which was then concentrated with 40% polyethylene glycol (Promega), as previously described^69^. HCT116 or HeLa cells were transduced by concentrated lentiviral particles and 5 µg ml^−1^ polybrene (Santa Cruz), followed by selection with 2 µg ml^−1^ puromycin (Gibco). The TRUB2 KD efficiency was confirmed by western blot analysis.

### Re-expression of RPUSD1 in the RPUSD1-KO cells

The pcDNA3.1-FLAG-RPUSD1 construct was generated by replacing the METTL3 sequence in the pcDNA3.1-FLAG-METTL3 plasmid (Addgene, 160250; https://www.addgene.org/160250/)^70^ with the open reading frame of WT *RPUSD1*. The pcDNA3.1-FLAG-RPUSD1-D67A construct was generated using Q5 Site-Directed Mutagenesis Kit (NEB) and oligonucleotides listed in Supplementary Table 2. All construct sequences were verified by Sanger sequencing. RPUSD1-KO cells were transfected with the specified construct using Lipofectamine 3000 Transfection Reagent (Invitrogen). Expression of each construct was verified by western blot.

### Western blot

WT, PUS-KO and PUS-KD HCT116 cells were lysed in RIPA buffer (0.1% SDS, 400 mM NaCl, 1 mM EDTA, 50 mM Tris–HCl and 1% Triton X-100) supplemented with protease inhibitor cocktail (Thermo). Protein samples were quantified using Pierce BCA Protein Assay Kit (Thermo) according to the manufacturer’s protocol. Fifty micrograms of total protein extract were incubated at 95 °C and separated on 10–12% acrylamide gel and transferred to Trans-Blot Turbo Midi 0.2 µm nitrocellulose membrane (Bio-Rad). Membranes were then blocked with 5% bovine serum albumin in 1× Tris-buffered saline solution with Tween 20, followed by incubation with the primary antibody either overnight at 4 °C or for 1 h at room temperature. The following primary antibodies were used: RPUSD1 antibody (Invitrogen, PA5-59448, lot R37969; 1:1,000 dilution), RPUSD2 antibody (Proteintech, 25707-1-AP, lot 00057317; 1:1,000 dilution), RPUSD3 antibody (Santa Cruz, sc-393209, lot 10413; 1:500 dilution), RPUSD4 antibody (Sigma, HPA039689, lot A118277; 1:1,000 dilution), PUS10 antibody (Abcam, ab313622, lot 1059517-4; 1:1,000 dilution), TRUB2 antibody (Proteintech, 19891-1-AP, lot 00076382; 1:1,000 dilution), PUS3 antibody (Proteintech, 17248-1-AP, lot 00099351; 1:1,000 dilution), PUSL1 antibody (Sigma, HPA032057, lot R32031; 1:1,000 dilution), and anti-vinculin antibody (Invitrogen, 700062, lot 2616511, clone number 42H89L44; 1:3,000 dilution). Anti-rabbit (Cell Signaling, 7074S, lot 33; 1:5,000 dilution) or anti-mouse (Cell Signaling, 7076S, lot 36; 1:5,000 dilution) antibody was used as the secondary antibody. Blots were visualized using Clarity ECL Substrate (Bio-Rad).

### RNA isolation

Total RNA was isolated using Quick-RNA Miniprep Kit (Zymo) according to the manufacturer’s protocol. Ribo^−^ RNA was isolated using RiboMinus Eukaryote System v2 (Invitrogen) according to the manufacturer’s protocol.

### BACS for Ψ detection

BACS and control libraries were constructed as previously described^18^. In brief, RNA was fragmented by NEBNext Magnesium RNA Fragmentation Module according to the manufacturer’s protocol. The fragmented RNA was 3′-end repaired using T4 PNK (NEB) and then ligated to RNA adapter (5′-/5rApp/AGATCGGAAGAGCGTCGTG/3SpC3/-3′) using T4 RNA Ligase 2, truncated KQ (NEB). Excess adapters were digested using 5′-deadenylase (NEB) and RecJ_f_ (NEB). For BACS, RNA was treated with 250 mM 2-bromoacrylamide (Enamine) in 625 mM phosphate buffer (pH 8.5) at 85 °C for 30 min. Both treated and control RNA were then reverse transcribed using reverse transcription (RT) primer (5′-ACACGACGCTCTTCCGATCT-3′) and Maxima H^−^ Reverse Transcriptase (Thermo). Excess RT primers were digested using Exo I (NEB). RNA was hydrolysed by sodium hydroxide (Sigma) and then neutralized by HCl (Sigma). The cDNA was ligated to cDNA adapter (5′-/5Phos/NNNNNNAGATCGGAAGAGCACACGTCTG/3SpC3/-3′) using T4 RNA Ligase 1, high concentration (NEB). The ligated cDNA was finally amplified with NEBNext Multiplex Oligos for Illumina (96 Unique Dual Index Primer Pairs) and NEBNext Ultra II Q5 Master Mix according to the manufacturer’s protocol. The PCR products were purified with 0.8× AMPure XP beads. BACS and control libraries were sequenced on a NextSeq 2000 (60-bp paired end reads) with no PhiX added.

To process multiple samples in one batch, multiplex BACS can be performed using barcoded RNA adapters as previously described^71^.

### Data preprocessing

Raw sequencing reads were processed by Cutadapt (v.4.9)^72^ to remove low-quality bases (-q 20) and short reads (-m 18), as well as to trim adaptors. 6-mer unique molecular identifiers (UMIs) were extracted by UMI-tools extract (v.1.0.1)^73^ and used for deduplication. Paired reads were then merged into single reads using fastp (v.0.23.2)^74^.

### Read alignment

Cleaned reads were first mapped to synthetic spike-ins and rRNA references using bowtie2 (v.2.4.4)^75^. The unaligned reads were subsequently mapped to tRNA references. High-confidence human tRNA sequences (hg38) were downloaded from GtRNAdb^76^. Only non-redundant tRNA sequences were kept and appended with a 3′-CCA end.

The aligned reads were then filtered and sorted using SAMtools (v.1.16.1)^77^. For synthetic spike-ins and rRNAs, only reads with MAPQ ≥10 were kept. For tRNAs, only reads with MAPQ ≥1 were kept. Deduplication was performed using UMI-tools dedup (v.1.0.1)^73^. Reads with correct strand information were kept. Finally, mutations were counted by SAMtools mpileup (v.1.16.1)^77^ and cpup (v.0.1.0) (https://github.com/y9c/cpup).

### Calling PUS-dependent Ψ sites

High-confidence Ψ sites in tRNAs were called on the basis of the following criteria: (1) coverage ≥50 in both BACS and control libraries; (2) background conversion rate ≤0.01 or T-to-C mutation counts ≤2 in control libraries; (3) background T-to-R (R = A or G) mutation ratio ≤0.10 in control libraries; (4) Ψ modification level ≥0.10 in cy-tRNA and ≥0.05 in mt-tRNA; (5) a *P* value was calculated for each site using the motif-specific false-positive rates and then adjusted following the Benjamini–Hochberg procedure; the adjusted *P* value is required to be <0.001. Only Ψ sites identified in expressed cy-tRNA isodecoders were reported. To assess PUS dependency, Ψ sites with coverage ≥20 in both KO replicates were used.

### Downstream analysis

The RNA structures were visualized using r2r (v.1.0.6)^78^. Sequence logos were generated using ggseqlogo (v.0.2)^79^. Differential sequence logos were generated using DiffLogo (v.2.32.0)^80^.

### Published data

Related published data were downloaded from the Gene Expression Omnibus (GEO) database: TRUB1-KO, PUS7-KO and PUS1-KO HeLa cells (GSE241849)^18^.

### Statistics and reproducibility

For PUS-KO and PUS-KD clones, two or three biologically independent replicates were used. Paired, two-tailed *t*-tests were used to calculate the statistical significance of differences between groups. Data distribution was assumed to be normal, but this was not formally tested. Individual data points were shown in all box plots. Statistical test and data visualization were performed by R (v.4.0.3).

No statistical method was used to predetermine sample sizes, but our sample sizes are similar to those reported in our previous publication^18^. No data were excluded from the analyses. The experiments were not randomized. The investigators were not blinded to allocation during experiments and outcome assessment. Data collection and analysis were not performed blind to experimental conditions, as consistency in cell culture, sample preparation, reagents, and experimental settings was necessary.

In certain cases, the *n* number is less than 3 as it represents the number of high-confidence Ψ sites in the specific positions. In Fig. 1b, *n* < 3 as *n* = 1 represents the only Ψ31 site in mt-tRNA. In Fig. 3a, *n* < 3 as *n* = 1 represents one Ψ20 site and one Ψ25 site in mt-tRNA; results are adapted from ref. ^18^. In Fig. 4a, *n* < 3 as *n* = 1 represents one Ψ50 site in mt-tRNA; results are adapted from ref. ^18^. In Fig. 4b, *n* < 3 as *n* = 1 represents one Ψe13 site in cy-tRNA.

### Reporting summary

Further information on research design is available in the Nature Portfolio Reporting Summary linked to this article.

## Online content

Any methods, additional references, Nature Portfolio reporting summaries, source data, extended data, supplementary information, acknowledgements, peer review information; details of author contributions and competing interests; and statements of data and code availability are available at 10.1038/s41556-025-01803-w.

## Supplementary information

Supplementary Information Supplementary Figs. 1–3.

Reporting Summary

Supplementary Tables 1–6 Supplementary Tables 1–6.

## Source data

Source Data Fig. 1 Statistical source data.

Source Data Fig. 2 Statistical source data.

Source Data Fig. 3 Statistical source data.

Source Data Fig. 4 Statistical source data.

Source Data Fig. 6 Statistical source data.

Source Data Extended Data Fig. 1 Unprocessed western blots.

Source Data Extended Data Fig. 2 Unprocessed western blots.

Source Data Extended Data Fig. 3 Unprocessed western blots.

Source Data Extended Data Fig. 6 Unprocessed western blots.

Source Data Extended Data Fig. 6 Statistical source data.

Source Data Extended Data Fig. 7 Statistical source data.

Source Data Extended Data Fig. 8 Statistical source data.

## Data Availability

All sequencing data are available at the GEO database (accession GSE285932). All relevant additional data have been published with this Resource, either as part of the main text or in the Supplementary Information. Source data are provided with this paper. All other data supporting the findings of this study are available from the corresponding author on reasonable request.

## Code availability

The analysis scripts are available via GitHub at https://github.com/lkong888/.
