# Supplementary material for: A comprehensive tRNA pseudouridine map uncovers targets dependent on human stand-alone pseudouridine synthases
Source: Nat Cell Biol. 2025 Oct 24;27(12):2186–97. doi: 10.1038/s41556-025-01803-w (PMC12716993; doi:10.1038/s41556-025-01803-w)
Supplement: Supplementary file 1 — Supplementary Figs. 1–3. [file 41556_2025_1803_MOESM1_ESM.pdf]

nature cell biology

Supplementary information https://doi.org/10.1038/s41556-025-01803-w

A comprehensive tRNA pseudouridine map uncovers targets dependent on human stand-alone pseudouridine synthases

In the format provided by the authors and unedited

Table of Contents

**Supplementary Fig. 1**	Comprehensive list of PUS-dependent Ψ sites across rRNAs and tRNAs
**Supplementary Fig. 2**	Summary of structural and sequence motifs for human stand-alone PUS targets.
**Supplementary Fig. 3**	Comparison of Ψ modifications within the anticodon between yeast and human.

PUS family	*E. coli*	*H. volcanii**	*S. cerevisiae*		*H. sapiens*	
TruD	TruD<br>tRNA<br>13	TruD<br>tRNA<br>13	Pus7<br>cy-tRNA<br>13, 35		PUS7<br>cy-tRNA<br>13, 20B, 35, 36, 50	mt-tRNA<br>50
					PUS7L<br>cy-tRNA<br>e12, e13, e1	
TruA	TruA<br>tRNA<br>38, 39, 40	TruA<br>tRNA<br>39	Pus3<br>cy-tRNA<br>38, 39	mt-tRNA<br>38, 39	PUS3<br>cy-tRNA<br>38, 39, 40	PUSL1<br>mt-tRNA<br>38, 39, 40
			Pus1<br>cy-tRNA<br>1, 26, 27, 28, 34, 36, 65, 67	Pus2<br>mt-tRNA<br>27, 28	PUS1<br>cy-tRNA<br>27, 28	mt-tRNA<br>20, 25, 27, 28, 66, 67, 68
TruB	TruB<br>tRNA<br>55		Pus4<br>cy-tRNA<br>55	mt-tRNA<br>55	TRUB1<br>cy-tRNA<br>55	mt-tRNA<br>55
						TRUB2<br>n/a
		Cbf5<br>rRNA	Cbf5<br>cy-rRNA		DKC1<br>cy-rRNA	
RluA	RluA<br>tRNA<br>32<br>23S rRNA<br>746		Pus8<br>cy-tRNA<br>32	Pus9<br>mt-tRNA<br>32	RPUSD2<br>cy-tRNA<br>31, 32, 34	mt-tRNA<br>31, 32
			Pus6<br>cy-tRNA<br>31	mt-tRNA<br>31		
					RPUSD1<br>cy-tRNA<br>30, 72	
	RluC<br>23S rRNA<br>955, 2504, 2580			Pus5<br>mt-21S rRNA<br>2819		RPUSD4<br>mt-16S rRNA<br>1397
						RPUSD3<br>n/a
	RluD<br>23S rRNA<br>1911, 1915, 1917					
	TruC<br>tRNA<br>65					
RsuA	RsuA<br>16S rRNA<br>516					
	RluB<br>23S rRNA<br>2605					
	RluE<br>23S rRNA<br>2457					
	RluF<br>23S rRNA<br>2604					
Pus10		Pus10<br>tRNA<br>54, 55			PUS10<br>cy-tRNA<br>54, 55	

Supplementary Fig. 1 | Comprehensive list of PUS-dependent Ψ sites across rRNAs and tRNAs. Results for *E. coli* and *S. cerevisiae* are adapted from ref.[1-3]. *Results for *H. volcanii* are not complete[4,5]. n/a, not applicable.

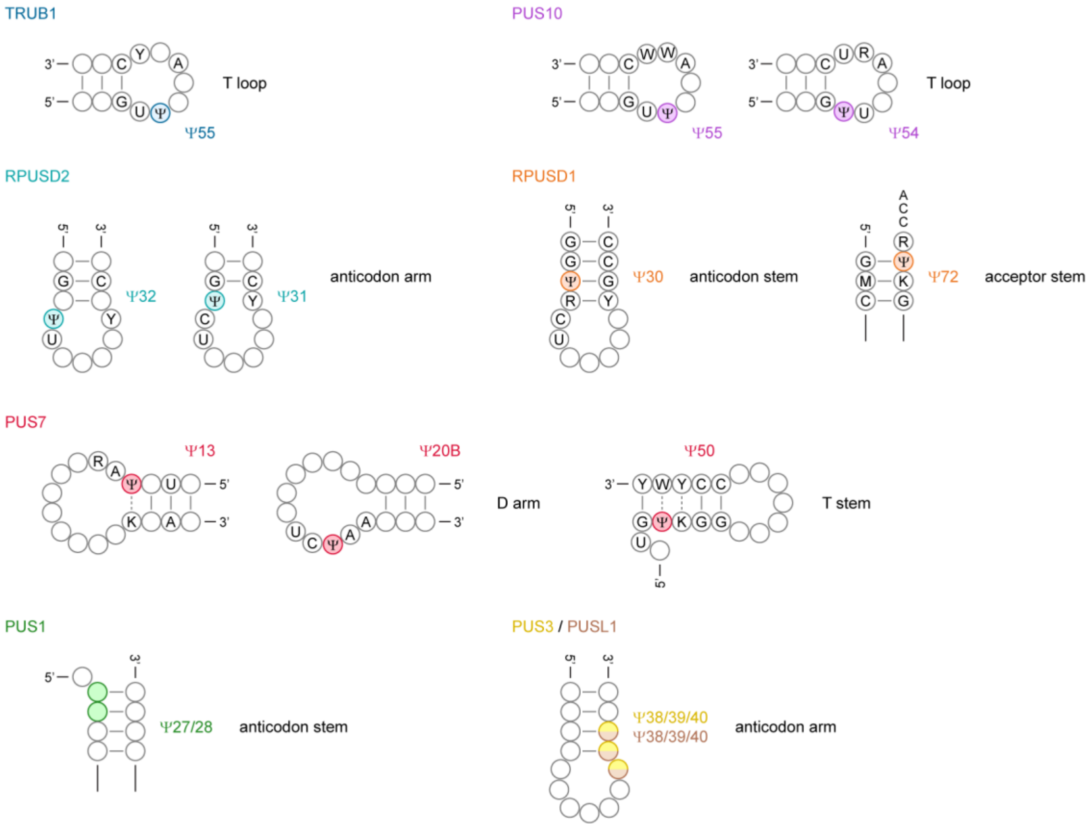

Supplementary Fig. 2 | Summary of structural and sequence motifs for human stand-alone PUS targets.

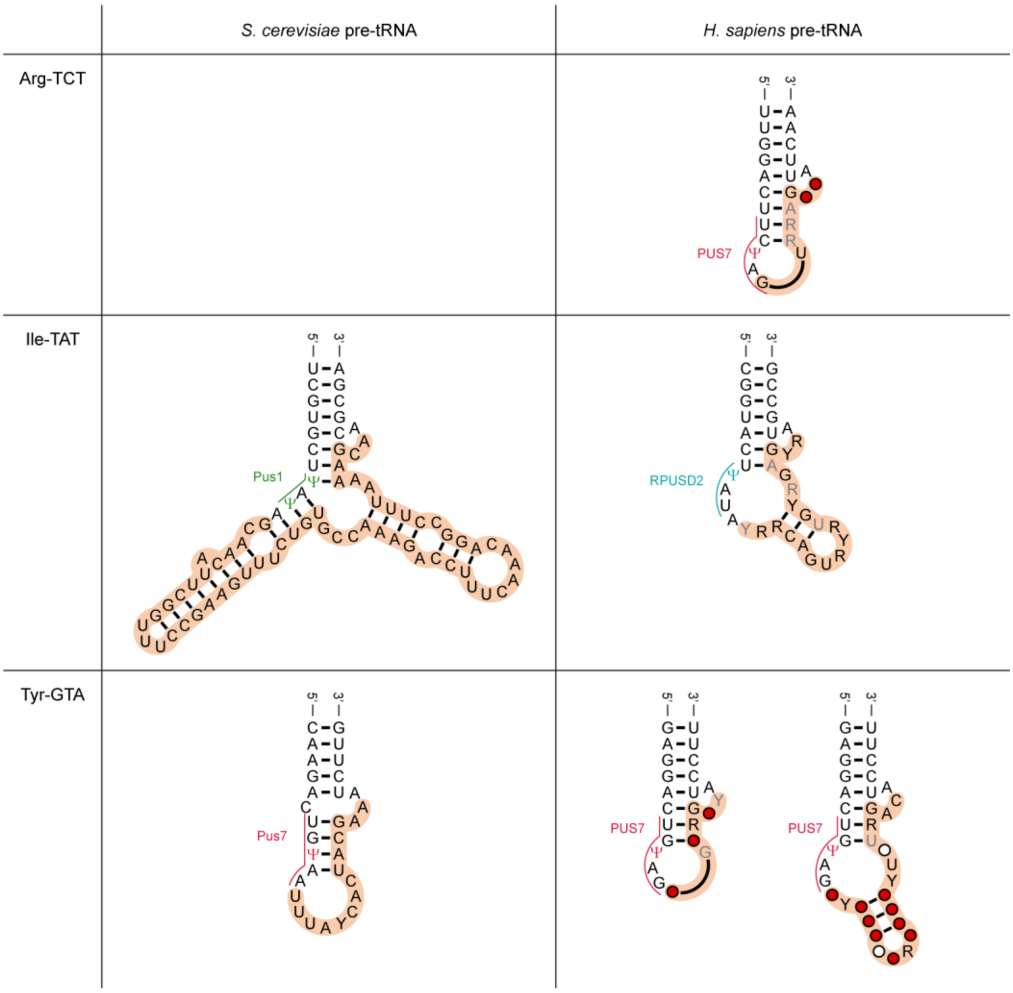

Supplementary Fig. 3 | Comparison of Ψ modifications within the anticodon between yeast and human. Intron sequences are labeled in orange.

References:

1. Hamma, T. & Ferré-D'Amaré, A. R. Pseudouridine synthases. *Chem. Biol.* **13**, 1125–1135 (2006).
2. Spenkuch, F., Motorin, Y. & Helm, M. Pseudouridine: still mysterious, but never a fake (uridine)! *RNA Biol.* **11**, 1540–1554 (2014).
3. Rintala-Dempsey, A. C. & Kothe, U. Eukaryotic stand-alone pseudouridine synthases – RNA modifying enzymes and emerging regulators of gene expression? *RNA Biol.* **14**, 1185–1196 (2017).
4. Grosjean, H., Gaspin, C., Marck, C., Decatur, W. A. & de Crécy-Lagard, V. RNomics and Modomics in the halophilic archaea *Haloferax volcanii*: identification of RNA modification genes. *BMC Genomics* **9**, 470 (2008).
5. Blaby, I. K. et al. Pseudouridine formation in archaeal RNAs: The case of *Haloferax volcanii*. *RNA* **17**, 1367–1380 (2011).
