# Supplementary material for: A comprehensive tRNA pseudouridine map uncovers targets dependent on human stand-alone pseudouridine synthases
Source: Nat Cell Biol. 2025 Oct 24;27(12):2186–97. doi: 10.1038/s41556-025-01803-w (PMC12716993; doi:10.1038/s41556-025-01803-w)
Supplement: Supplementary file 2 — Reporting Summary [file 41556_2025_1803_MOESM2_ESM.pdf]

Corresponding author(s): Chun-Xiao Song
Parinaz Mehdipour

Last updated by author(s): Sep 30, 2025

Reporting Summary

Nature Portfolio wishes to improve the reproducibility of the work that we publish. This form provides structure for consistency and transparency in reporting. For further information on Nature Portfolio policies, see our Editorial Policies and the Editorial Policy Checklist.

Statistics

For all statistical analyses, confirm that the following items are present in the figure legend, table legend, main text, or Methods section.

n/a	Confirmed	
☐	☒	The exact sample size (*n*) for each experimental group/condition, given as a discrete number and unit of measurement
☐	☒	A statement on whether measurements were taken from distinct samples or whether the same sample was measured repeatedly
☐	☒	The statistical test(s) used AND whether they are one- or two-sided<br>*Only common tests should be described solely by name; describe more complex techniques in the Methods section.*
☐	☒	A description of all covariates tested
☐	☒	A description of any assumptions or corrections, such as tests of normality and adjustment for multiple comparisons
☐	☒	A full description of the statistical parameters including central tendency (e.g. means) or other basic estimates (e.g. regression coefficient) AND variation (e.g. standard deviation) or associated estimates of uncertainty (e.g. confidence intervals)
☐	☒	For null hypothesis testing, the test statistic (e.g. *F*, *t*, *r*) with confidence intervals, effect sizes, degrees of freedom and *P* value noted<br>*Give P values as exact values whenever suitable.*
☒	☐	For Bayesian analysis, information on the choice of priors and Markov chain Monte Carlo settings
☐	☒	For hierarchical and complex designs, identification of the appropriate level for tests and full reporting of outcomes
☐	☒	Estimates of effect sizes (e.g. Cohen's *d*, Pearson's *r*), indicating how they were calculated

Our web collection on statistics for biologists contains articles on many of the points above.

Software and code

Policy information about availability of computer code

Data collection	Illumina NextSeq 2000 and BaseSpace
Data analysis	Paired-end reads were download as FASTQ from Illumina BaseSpace. Statistical analyses were performed in R (v.4.0.3). The details are described in the Methods section. The analysis scripts are available at https://github.com/lkong888/bacs.<br>Cutadapt (v.4.9), UMI-tools (v.1.0.1), fastp (v.0.23.2), bowtie2 (v.2.4.4), SAMtools (v.1.16.1), cpup (v.0.1.0), r2r (v.1.0.6), ggseqlogo (v.0.2), DiffLogo (v.2.32.0), R (v.4.0.3 & v.4.3.1).

For manuscripts utilizing custom algorithms or software that are central to the research but not yet described in published literature, software must be made available to editors and reviewers. We strongly encourage code deposition in a community repository (e.g. GitHub). See the Nature Portfolio guidelines for submitting code & software for further information.

Data

Policy information about availability of data

All manuscripts must include a data availability statement. This statement should provide the following information, where applicable:

- Accession codes, unique identifiers, or web links for publicly available datasets
- A description of any restrictions on data availability
- For clinical datasets or third party data, please ensure that the statement adheres to our policy

All sequencing data are available at the GEO database (accession: GSE285932). All relevant additional data have been published with the manuscript, either as part

of the main text or in the supplement. Source data have been provided in Source Data. All other data supporting the findings of this study are available from the corresponding author on reasonable request.

Reads were mapped to human rRNA and tRNA references:
Human rRNA sequences were downloaded from NCBI (NR_023363.1, NR_003285.3, NR_003286.4, NR_003287.4);
High-confidence human tRNA sequences (hg38) were downloaded from GtRNAdb (https://gtrnadb.ucsc.edu/).

Related published data were downloaded from the Gene Expression Omnibus (GEO) database: TRUB1-KO, PUS7-KO, and PUS1-KO HeLa cells (GSE241849).

Research involving human participants, their data, or biological material

Policy information about studies with human participants or human data. See also policy information about sex, gender (identity/presentation), and sexual orientation and race, ethnicity and racism.

Reporting on sex and gender	n/a
Reporting on race, ethnicity, or other socially relevant groupings	n/a
Population characteristics	n/a
Recruitment	n/a
Ethics oversight	n/a

Note that full information on the approval of the study protocol must also be provided in the manuscript.

Field-specific reporting

Please select the one below that is the best fit for your research. If you are not sure, read the appropriate sections before making your selection.

☒ Life sciences ☐ Behavioural & social sciences ☐ Ecological, evolutionary & environmental sciences

For a reference copy of the document with all sections, see nature.com/documents/nr-reporting-summary-flat.pdf

Life sciences study design

All studies must disclose on these points even when the disclosure is negative.

Sample size	No statistical methods were used to predetermine the sample size. All sample sizes were determined based on our prior experiences on similar experiments and published studies (Xu et al., Nat. Methods 2024).
Data exclusions	No data were excluded from the analyses.
Replication	Yes, as described in figure legends and Methods.
Randomization	Randomization was not relevant to this study. Controlling covariates was not necessary because experimental and control samples were processed in parallel.
Blinding	Blinding was not required because the results of measurement or analysis was not affected by knowledge of sample identities.

Reporting for specific materials, systems and methods

We require information from authors about some types of materials, experimental systems and methods used in many studies. Here, indicate whether each material, system or method listed is relevant to your study. If you are not sure if a list item applies to your research, read the appropriate section before selecting a response.

Materials & experimental systems

n/a	Involved in the study	
	☒	Antibodies
	☒	Eukaryotic cell lines
☒		Palaeontology and archaeology
☒		Animals and other organisms
☒		Clinical data
☒		Dual use research of concern
☒		Plants

Methods

n/a	Involved in the study	
☒		ChIP-seq
☒		Flow cytometry
☒		MRI-based neuroimaging

Antibodies

Antibodies used

RPUSD1 antibody (Invitrogen, PA5-59448, lot R37969; 1:1,000 dilution);
RPUSD2 antibody (Proteintech, 25707-1-AP, lot 00057317; 1:1,000 dilution);
RPUSD3 antibody (Santa Cruz, sc-393209, lot 10413; 1:500 dilution);
RPUSD4 antibody (Sigma, HPA039689, lot A118277; 1:1,000 dilution);
PUS10 antibody (Abcam, ab313622, lot 1059517-4; 1:1,000 dilution);
TRUB2 antibody (Proteintech, 19891-1-AP, lot 00076382; 1:1,000 dilution);
PUS3 antibody (Proteintech, 17248-1-AP, lot 00099351; 1:1,000 dilution);
PUSL1 antibody (Sigma, HPA032057, lot R32031; 1:1,000 dilution);
Anti-vinculin antibody (Invitrogen, 700062, lot 2616511, clone number 42H89L44; 1:3,000 dilution);
Anti-rabbit antibody (Cell Signaling, 7074S, lot 33; 1:5,000 dilution);
Anti-mouse antibody (Cell Signaling, 7076S, lot 36; 1:5,000 dilution).

Validation

RPUSD1 antibody (Invitrogen, PA5-59448; 1:1,000 dilution): https://www.thermofisher.com/antibody/product/RPUSD1-Antibody-Polyclonal/PA5-59448
Positive WB detected in HEK293T cells

RPUSD2 antibody (Proteintech, 25707-1-AP; 1:1,000 dilution): https://www.ptglab.com/products/RPUSD2-Antibody-25707-1-AP.htm
Positive WB detected in HEK-293, HeLa cells
Positive IHC detected in human colon cancer tissue, human breast cancer tissue

RPUSD3 antibody (Santa Cruz, sc-393209; 1:500 dilution): https://www.scbt.com/p/rpusd3-antibody-c-2
Positive WB detected in HEL 92.1.7, Hep G2 cells

RPUSD4 antibody (Sigma, HPA039689; 1:1,000 dilution): https://www.sigmaaldrich.com/GB/en/product/sigma/hpa039689
Positive WB detected in HEK293T cells
Antonicka et al., EMBO Rep. 2017

PUS10 antibody (Abcam, ab313622; 1:1,000 dilution): https://www.abcam.com/en-us/products/primary-antibodies/pus10-antibody-epr26272-66-ab313622
Positive WB detected in HEK293T, HepG2, HeLa, PC-3 cells

TRUB2 antibody (Proteintech, 19891-1-AP; 1:1,000 dilution): https://www.ptglab.com/products/TRUB2-Antibody-19891-1-AP.htm
Positive WB detected in HT-1080, HeLa, HepG2, K-562 cells
Positive IHC detected in human colon cancer tissue
Antonicka et al., EMBO Rep. 2017

PUS3 antibody (Proteintech, 17248-1-AP; 1:1,000 dilution): https://www.ptglab.com/products/PUS3-Antibody-17248-1-AP.htm
Positive WB detected in A549, HEK-293, HeLa, Jurkat cells
Qi et al., Anal. Chem. 2022

PUSL1 antibody (Sigma, HPA032057; 1:1,000 dilution): https://www.sigmaaldrich.com/GB/en/product/sigma/hpa032057
Positive WB detected in SCLC-21H cells
Busch et al., Cell Rep. 2019

Anti-vinculin antibody (Invitrogen, 700062; 1:3,000 dilution): https://www.thermofisher.com/antibody/product/Vinculin-Antibody-clone-42H89L44-Recombinant-Monoclonal/700062

Anti-rabbit antibody (Cell Signaling, 7074S; 1:5,000 dilution):
https://www.cellsignal.co.uk/products/secondary-antibodies/anti-rabbit-igg-hrp-linked-antibody/7074

Anti-mouse antibody (Cell Signaling, 7076S; 1:5,000 dilution):
https://www.cellsignal.co.uk/products/secondary-antibodies/anti-mouse-igg-hrp-linked-antibody/7076

Eukaryotic cell lines

Policy information about cell lines and Sex and Gender in Research

Cell line source(s)	HCT116 cells (#CCL-247) was obtained from ATCC. HeLa cells were gifted from Prof Peter J. Ratcliffe (University of Oxford) (originally obtained from ATCC, #CCL-2).
Authentication	HCT116 cell line was authenticated by ATCC STR profiling.
Mycoplasma contamination	All cell lines were negative for mycoplasma test.
Commonly misidentified lines (See ICLAC register)	No commonly misidentified cell lines were used.

Plants

Seed stocks	*Report on the source of all seed stocks or other plant material used. If applicable, state the seed stock centre and catalogue number. If plant specimens were collected from the field, describe the collection location, date and sampling procedures.*
Novel plant genotypes	*Describe the methods by which all novel plant genotypes were produced. This includes those generated by transgenic approaches, gene editing, chemical/radiation-based mutagenesis and hybridization. For transgenic lines, describe the transformation method, the number of independent lines analyzed and the generation upon which experiments were performed. For gene-edited lines, describe the editor used, the endogenous sequence targeted for editing, the targeting guide RNA sequence (if applicable) and how the editor was applied.*
Authentication	*Describe any authentication procedures for each seed stock used or novel genotype generated. Describe any experiments used to assess the effect of a mutation and, where applicable, how potential secondary effects (e.g. second site T-DNA insertions, mosiacism, off-target gene editing) were examined.*
