# Supplementary material for: A comprehensive tRNA pseudouridine map uncovers targets dependent on human stand-alone pseudouridine synthases
Source: Nat Cell Biol. 2025 Oct 24;27(12):2186–97. doi: 10.1038/s41556-025-01803-w (PMC12716993; doi:10.1038/s41556-025-01803-w)

Extended Data Fig. 1b. RPUSD1-KO

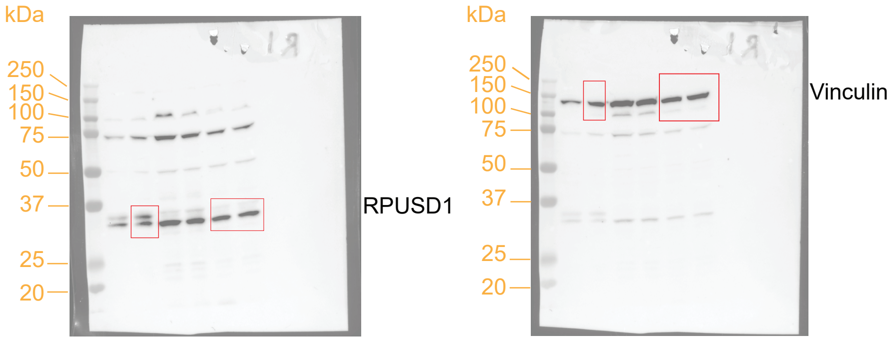


Extended Data Fig. 1b. RPUSD2-KO

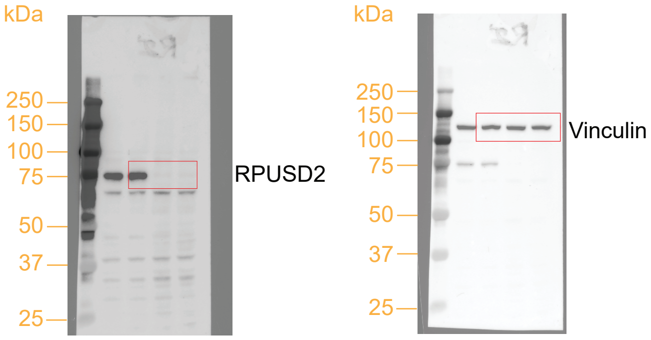


Extended Data Fig. 1b. RPUSD3-KO

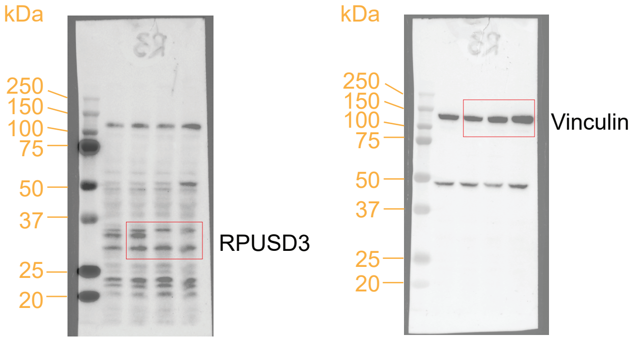


Extended Data Fig. 1b. RPUSD4-KD

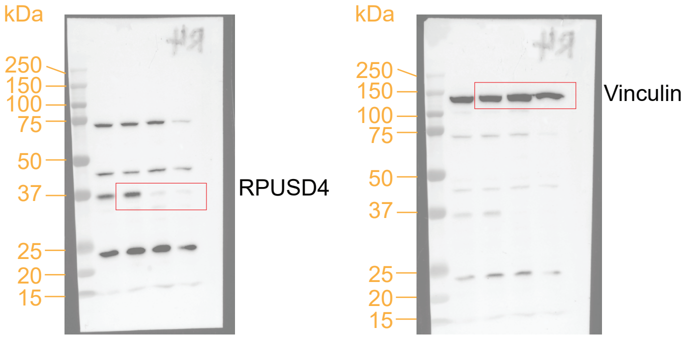

Supplement: Supplementary file 9 — Unprocessed western blots. [file 41556_2025_1803_MOESM9_ESM.pdf]
