# Supplementary figures and images for: A comprehensive tRNA pseudouridine map uncovers targets dependent on human stand-alone pseudouridine synthases
Source: Nat Cell Biol. 2025 Oct 24;27(12):2186–97. doi: 10.1038/s41556-025-01803-w (PMC12716993; doi:10.1038/s41556-025-01803-w)

Extended Data Fig. 2b. PUS10-KO

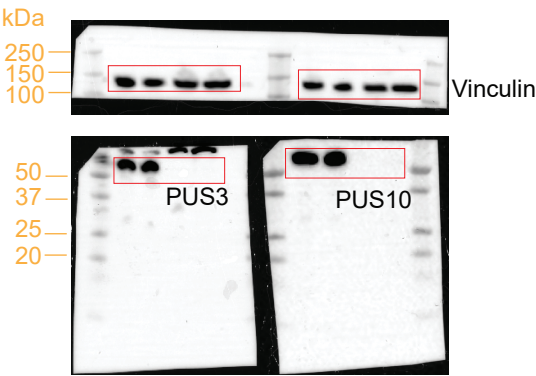

Extended Data Fig. 2c. TRUB2-KD HCT116

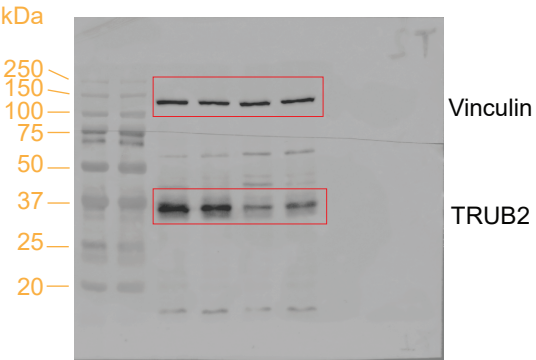

Extended Data Fig. 2c. TRUB2-KD HeLa

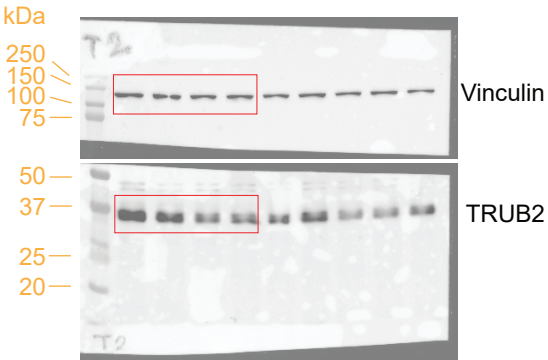

Supplement: Supplementary file 10 — Unprocessed western blots. [file 41556_2025_1803_MOESM10_ESM.pdf]

Extended Data Fig. 3b. PUS3-KO

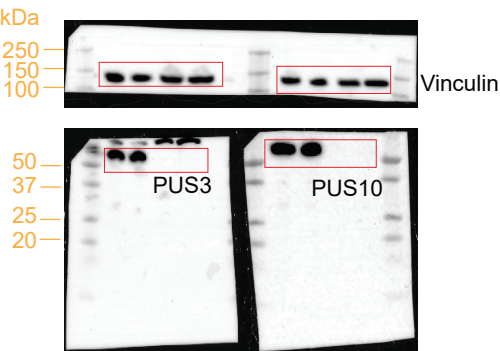

Extended Data Fig. 3b. PUSL1-KO

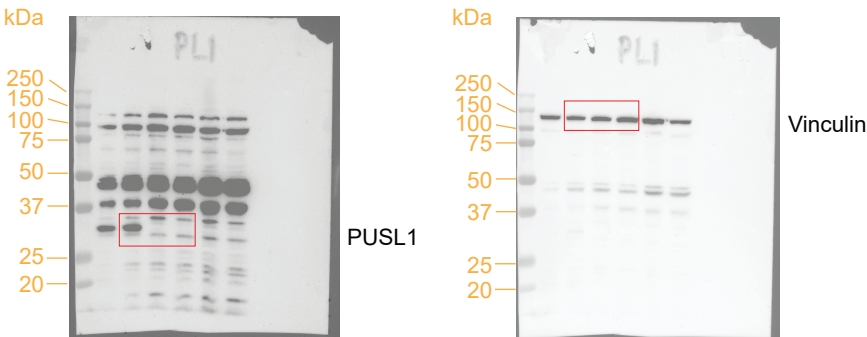

Supplement: Supplementary file 11 — Unprocessed western blots. [file 41556_2025_1803_MOESM11_ESM.pdf]

Extended Data Fig. 6a. RPUSD1-rescue

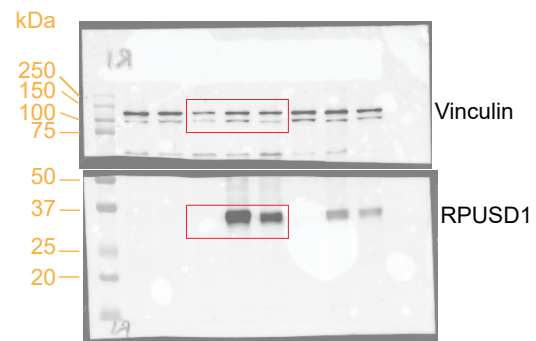

Supplement: Supplementary file 12 — Unprocessed western blots. [file 41556_2025_1803_MOESM12_ESM.pdf]
